# Targeting PTGDS Promotes ferroptosis in peripheral T cell lymphoma through regulating HMOX1-mediated iron metabolism

**DOI:** 10.1038/s41416-024-02919-w

**Published:** 2024-12-20

**Authors:** Shunfeng Hu, Bingyu Liu, Juanjuan Shang, Qianqian Guo, Tiange Lu, Xiaoli Zhou, Xiangxiang Zhou, Xin Wang

**Affiliations:** 1 Department of Hematology, Shandong Provincial Hospital, Shandong University, Jinan, Shandong 250021 China; 2 Department of Hematology, Shandong Provincial Hospital Affiliated to Shandong First Medical University, Jinan, Shandong 250021 China; 3 Taishan Scholars Program of Shandong Province, Jinan, Shandong 250021 China; 4 Branch of National Clinical Research Center for Hematologic Diseases, Jinan, Shandong 250021 China; 5 National Clinical Research Center for Hematologic Diseases, the First Affiliated Hospital of Soochow University, Suzhou, 251006 China

**Keywords:** T-cell lymphoma, Protein-protein interaction networks, Cell death, Prognostic markers

## Abstract

**Background:**

Peripheral T cell lymphoma (PTCL) is characterized by high heterogeneity, strong aggressiveness, and extremely poor prognosis. Ferroptosis, a novel form of programmed cell death, has been involved in tumor development and targeting ferroptosis holds great potential for tumor therapy.

**Methods:**

Lentiviral transfection was performed to regulate gene expression, followed by Tandem mass tag (TMT)-mass spectrometry and RNA-sequencing. Tumor xenograft models were established for in vivo experiments.

**Results:**

High expression of prostaglandin D2 synthase (PTGDS) was closely associated with poor prognosis of PTCL patients. PTGDS knockdown and AT56 treatment significantly inhibited the progression of PTCL through regulating cell viability, proliferation, apoptosis, cell cycle and invasion in vitro and in vivo. We further revealed that targeting PTGDS promoted ferroptosis process and enhanced the sensitivity of PTCL cells to ferroptosis inducers Sorafenib in vitro and in vivo. Mechanically, PTGDS interacted with heme-degrading enzymes HMOX1, and targeting PTGDS increased the level of iron and induced ferroptosis in PTCL through promoting HMOX1-mediated heme catabolism and ferritin autophagy process. Through the construction of H25A mutation, the specific gene site of HMOX1 corresponding to its role was identified.

**Conclusions:**

Taken together, our findings firstly identified that targeting PTGDS promotes the ferroptosis in PTCL through regulating HMOX1-mediated iron metabolism, and highlighted novel therapeutic strategies to improve the efficacy of ferroptosis-targeted therapy in PTCL patients.

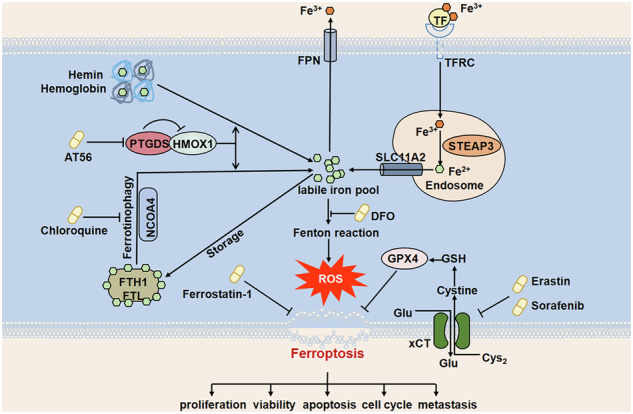

## Introduction

Peripheral T cell lymphoma (PTCL) is a group of aggressive tumors derived from mature T cells and natural killer cells, with high heterogeneity, strong aggressiveness and extremely poor prognosis [1]. With the development of novel comprehensive treatment, including immune-chemotherapy and cell therapy, the prognosis of PTCL patients has been greatly improved [2, 3]. However, with its unknown pathogenesis and prolonged stagnation of first-line therapy, the survival rates of PTCL patients are still below 30%. Therefore, identifying more precise molecular targets and efficiently targeted intervention are still needed to improve the clinical benefit of PTCL patients.

Ferroptosis is a recently identified form of regulated cell death, which displays distinct features in terms of morphology, biochemistry, and molecular mechanisms [4]. Ferroptosis is characterized by iron dependence, reactive oxygen species (ROS) accumulation and lipid peroxidation [5]. As an iron-dependent cell death, iron metabolism, including absorption, transport, storage and release, plays an essential role in ferroptosis regulation, which is regulated by complex gene regulatory networks [6]. Although ferroptosis has been initially recognized to be distinct from other types of cell death [4], increasing evidence has revealed the important role of selective autophagy in ferroptosis induction [7, 8], including ferritinophagy, lipophagy, clockophagy, and chaperone-mediated autophagy.

Ferroptosis dysfunction has been found to be involved in the development of several tumors [9], and numerous novel therapies targeting ferroptosis hold great potential as promising treatment strategies for tumor patients [10]. Therefore, increasing interest has been focused on the identification of regulatory networks and molecular mechanisms regulating ferroptosis in tumor development. However, only little progress has been achieved in the regulating network and clinical application of ferroptosis in PTCL development.

We have reported that prostaglandin D2 synthase (PTGDS) promotes the tumorigenesis of diffuse large B-cell lymphoma (DLBCL) by MYH9-mediated regulation of Wnt-β-catenin-STAT3 signaling [11], and previous studies found that PTGDS was involved in tumor development through regulating MAPK pathway [12], PPARγ [13] and STAT3 phosphorylation [14]. Moreover, recent studies have revealed that the downstream molecules and signaling pathways of PTGDS played regulatory roles in ferroptosis process [15–19]. Therefore, whether PTGDS serves as the key factor in ferroptosis process of PTCL cells has attracted our great interest. Here, our present study firstly revealed that high expression of PTGDS was closely associated with poor prognosis in PTCL patients. In-vitro and in-vivo studies found that targeting PTGDS could significantly inhibit the progression of PTCL through promoting ferroptosis process and iron metabolism, which was mediated by heme oxygenase 1 (HMOX1)-mediated heme degradation and ferritin autophagy. Point mutation confirmed the specific gene site of HMOX1 corresponding to its function while targeting PTGDS in PTCL. Collectively, our study firstly identified the regulatory role and molecular mechanism of PTGDS in ferroptosis process and iron metabolism, providing promising strategies to facilitate ferroptosis-targeted therapy in the treatment of PTCL patients.

## Materials and methods

### Patient samples

Ethical approval was obtained from Medical Ethical Committee of Shandong Provincial Hospital and written informed consent was obtained from each patient in accordance with the Declaration of Helsinki. The diagnoses of all patients were histologically confirmed based on the 2022 WHO classification system [20] and all patients received standard treatment according to international guidelines. The paraffin-embedded specimens were collected from 159 newly diagnosed PTCL patients and 38 reactive lymphadenitis patients from Jan 2008 to Dec 2023. Besides, serum and peripheral blood mononuclear cells (PBMCs) were also isolated from PTCL patients and healthy donors, and T cells were purified from freshly isolated PBMCs by sheep red blood cell rosette method according to previous protocols [21]. Detailed information related to PTCL patients were shown in Supplementary tables 2 and 4.

### Cell lines and reagents

We purchased Karpas299, Myla3676 and Jurkat cells from ATCC and cultured them in RPMI 1640 (Gibco, CA, USA) with 10% fetal bovine serum (HyClone, UT, USA), 1% penicillin/streptomycin mixture, and 2 mM glutamine. Cells were cultured under humidified air containing 5% CO_2_ at 37 °C. The examination of Short tandem repeat (STR) and mycoplasma infection was periodically performed. AT56 was obtained from Cayman (13160, MI, USA). Sorafenib (S7397), Erastin (S7242) and Ferrostatin-1 (S7243) were purchased from Selleck Chemicals (TX, USA). Deferoxamine (DFO, D-9533) and Chloroquine (C6628) were bought from Sigma-Aldrich (MO, USA).

### Immunohistochemistry (IHC) and hematoxylin–eosin (HE) staining

IHC and HE staining were performed according to standard methods. Results of IHC were independently assessed by two observers at two different time points, who were blinded to patients’ information. IHC score was computed by the following formula: IHC score = proportion score (0, none; 1, 1%~25%; 2, 26%~50%; 3, 51%~75%; and 4, 76%~100%) × intensity score (0, negative; 1, weak; 2, moderate; and 3, strong). Scores of 0–7 were defined as negative expression, 8–12 as positive expression. The primary antibodies included PTGDS (ab182141, Abcam) and Ki67 (27309-1-AP, Proteintech Group).

### Elisa assay

Peripheral blood of 76 PTCL patients and 31 healthy volunteers was collected followed by subsequent centrifugation to isolate serum. The concentration of soluble PTGDS protein in serum was evaluated using ELISA Kit (MB-0408, MBBIOLOGY, China)

### Cell transfection

The construction of lentivirus to overexpress PTGDS, knockdown PTGDS and knockdown HMOX1 or control was performed from Genechem (Shanghai, China). Sequences were listed as follows: sh-PTGDS#1, CAGGGCTGAGTTAAAGGAGAA; sh-PTGDS#2, GATAAGTGCATGACGGAACAA; sh-HMOX1#1, TGCCAGTGCCACCAAGTTCAA; sh-HMOX1#2, ATGGGTCCTTACACTCAGCTT; sh-HMOX1#3, AGGCAGAGAATGCTGAGTTCA. Lentiviral transfection was performed following the manufacturer’s instructions, and 5 μg/mL puromycin (AMRESCO, USA) was used to screen cells with stable transfection. In addition, the plasmids (HA-HMOX1-WT, HA-HMOX1-H25A) were synthesized by Biosune (Shanghai, China), and plasmids were transfected into cells through Lipofectamine 2000 (Invitrogen, CA, USA) according the manufacturer’s instructions.

### Cell proliferation, viability, and invasion assay

Counted PTCL cells (1 × 10^4^ cells / 100 μL / well) were seeded in triplicates in 96-well plates. Cell Counting Kit-8 (CCK-8) assay kit (CK04, Dojindo, Japan) was used to evaluate cell proliferation via Multiskan GO Microplate Reader (Thermo Scientific, IL, USA). Cell viability was detected through CellTiter-Glo Luminescent assays (G7570, Promega Corporation, WI, USA) and microplate luminometer (Centro XS3 LB960, Berthold Technologies, Stuttgart, Germany). Cell invasion was measured using 24-well transwells (8.0 μm, Corning, USA) precoated with matrigel.

### Flow cytometry analysis

To evaluate cell cycle and cell apoptosis, Propidium iodide (PI)/RNase Staining Buffer (550825, BD Biosciences, MA, USA) and Annexin V-PE/7-aminoactinomycin D (7AAD) (559763, BD Biosciences) was used, and fluorescence was detected using Navios Flow Cytometer (Beckman Coulter, CA, USA). C11-BODIPY (581/591) (D3861, Invitrogen) was used to evaluate the level of lipid ROS production in PTCL cells. Data were processed using FlowJo (FlowJo, LLC) software.

### Western blotting

Protein extraction and western blotting were performed as previously described [11]. The primary antibodies included HMOX1 (66743-1-Ig, Proteintech Group), NRF2 (66504-1-Ig, Proteintech Group), NCOA4 (39896, Proteintech Group), antibodies from Abcam, including PTGDS (ab182141), KEAP1 (ab227828), ACSL4 (ab155282), xCT (ab175186), GPX4 (ab125066), FTH1 (ab75972), FTL (ab109373), Ferritin (ab75973), P62 (ab109012), Beclin 1 (ab207612), LC3B (ab192890), and antibodies bought from Cell Signaling Technology (Beverly, USA), including c-myc (18583), Cyclin D1 (2922), CDK4 (12790), P21 (2947), P27 (3686), caspase 3 (9662), caspase 9 (9508), PARP (9532), Bax (5023), zeb1(3396), vimentin (5741) and HA (3724). β-tubulin (86298), β-actin (4967) and GAPDH (97166) were used as the internal reference.

### RNA-sequencing and quantitative tandem mass tags (TMT) mass spectrometry

Karpas cells treated with AT56 (75 μM for 48 h) or DMSO, and each group comprised three replicates. RNA-sequencing and quantitative TMT proteomics were performed by Novogene (Beijing, China). In RNA-sequencing, total RNA was extracted according to standard protocols [11]. Total amounts and integrity of RNA were assessed using the RNA Nano 6000 assay kit of the Bioanalyzer 2100 system (Agilent Technologies, CA, USA), and sequencing libraries were prepared as recommended by the manufacturer, which was quantified using Qubit 2.0 Fluorometer. Further sequencing was performed by the Illumina NovaSeq 6000. In quantitative TMT proteomics, to extract total proteins, Karpas cells were lysed with lysis buffer (pH 8.5), followed by ultrasonication on ice. The lysate was centrifuged at 12,000 g for 15 min at 4 °C and the supernatant was reduced with 10 mM DTT for 1 h at 56 °C, and subsequently alkylated with sufficient iodoacetamide for 1 h at room temperature in the dark. The examination of quality and quantity of total proteins was performed using BSA kit and SDS-PAGE gel electrophoresis. Proteins were digested into peptides and labeled using TMT labeling reagent. Further proteomics analyses were performed using EASY-nLC^TM^ 1200 UHPLC system (Thermo Fisher), Q Exactive^TM^ HF-X mass spectrometer (Thermo Fisher) and ion source of Nanospray Flex^TM^ (ESI). Protein identification was conducted using Proteome Discoverer 2.4 (PD 2.4, Thermo), and protein quantification results were statistically analyzed by t-test. Data were analyzed using R software.

### Iron assay

Ferrous iron level was determined using the iron assay kit (ab83366, Abcam) according to the manufacturer’s instructions. The absorbance values at 593 nm were measured, and the standard curves were drawn according to the absorbance values of standard samples. Ferrous iron level of samples was measured according to absorbance values and standard curves.

### Immunofluorescence assays and confocal microscopy

Confocal microscopy was performed using Leica TCS SP8 MP confocal microscope system (Germany). The primary antibodies included HMOX1 (66743-1-Ig, Proteintech Group) and PTGDS (ab182141, Abcam).

### Molecular docking

We obtained the structures of proteins from the PDB database (https://www.rcsb.org/). Molecular docking was performed using the HDOCK online server (http://hdock.phys.hust.edu.cn/), which could analyze different conformations of protein-protein docking, evaluate binding activities under different conformations, and identify amino acid residues with interaction distance less than 5 Å. Molecular docking pattern diagram was drawn using the PyMOL (version 4.6.0) software. Confidence score was used to evaluate the binding potential between proteins.

### Co-immunoprecipitation (Co-IP)

Total proteins were extracted from PTCL cells using Co-IP lysis buffer, which were incubated with 1–3 μg primary antibody at 4 °C overnight. The primary antibodies included PTGDS (ab182141, Abcam), HMOX1 (66743-1-Ig, Proteintech Group) and HA (2367, CST).

### In-vivo xenograft tumor models

4-week-old female severe combined immunodeficiency (SCID) beige mice were bought from Weitong Lihua Laboratory Animal Center (Beijing, China) and raised in specific pathogen-free (SPF) environment. All animal experiments were approved by Animal Care and Research Advisory Committee of Shandong Provincial Hospital. Simple randomization was performed and no blinding method was used. 1 × 10^7^ Karpas299 cells (untransfected, sh-Control, sh-PTGDS) were subcutaneously injected into the right hind legs of mice. AT56 was orally administered by gavage (80 mg/kg) while Sorafenib was intraperitoneally administered (80 mg/kg). In-vivo small animal imaging system (Berthold Technologies, Germany) was used to evaluate the growth and metabolic activity of tumor.

### Statistical analysis

All statistics were computed with the SPSS 23.0 software (SPSS Inc, USA). Data were presented as mean ± standard deviation (SD) from at least three independent experiments and all data were examined for normality and homogeneity of variance. Continuous variables were analyzed by using Students *t*-test and non-parametric tests, and categorical variables using the χ2 test. Survival analysis was determined using the Kaplan-Meier method with log-rank test. There was no statistical method used to determine the sample size in our study. *P* < 0.05 was considered as statistically significant (*p < 0.05, **p < 0.01, ***p < 0.001).

## Results

### High expression of PTGDS was associated with tumor progression of PTCL

**Fig. 1:**
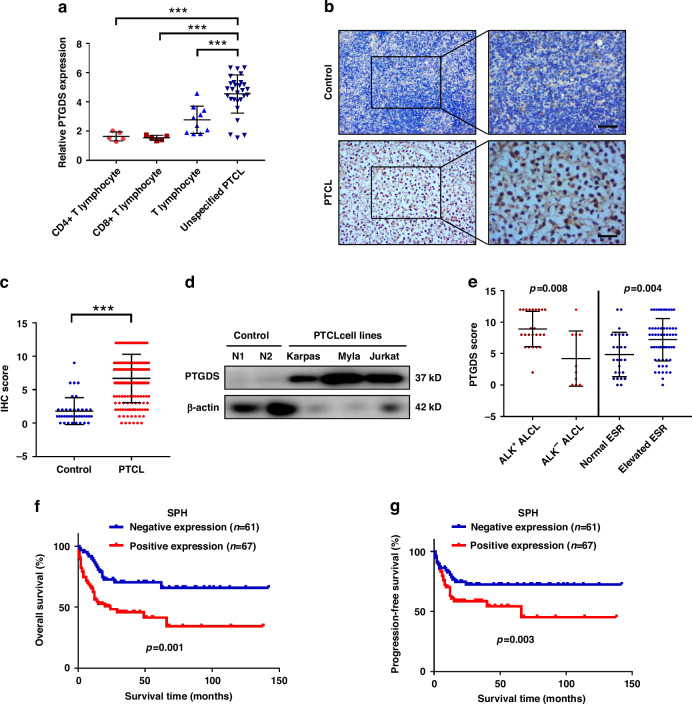
High expression of PTGDS was associated with tumor progression of PTCL. **a** The expression of PTGDS mRNA in tissue from PTCL patients was higher than that in normal T cells based on Oncomine database. **b** Representative pictures of immunohistochemical staining from PTCL tissue and control sample. Bar = 40 μm. **c** Statistic analysis showed the increased expression of PTGDS protein in PTCL tissue (*n* = 159) in comparison with control samples (*n* = 38). **d** Results of western blotting showed that the expression level of PTGDS protein was higher in PTCL cell lines than that in normal T cells. **e** IHC score of PTGDS was found to be associated with clinical features in PTCL patients. **f**, **g** Kaplan–Meier survival analysis revealed that the positive expression of PTGDS was associated with worse OS and PFS in PTCL patients. Data are shown as the mean ± SD. ***p < 0.001.

Firstly, we evaluated the expression level of PTGDS based on data from Oncomine database, and also found the higher expression of PTGDS in PTCL in comparison with normal T cells (*p* < 0.001) (Fig. 1a). Then, analysis based on GSE6338 database verified the high expression of PTGDS in PTCL patients (Supplementary Fig. S1A–C). Moreover, results from IHC assay showed that the expression level of PTGDS protein was higher in PTCL tissue (*n* = 159) than that in control samples (*n* = 38) (Fig. 1b, c). Similarly, compared with normal T cells from PBMCs of healthy donors, the expression of PTGDS was increased in PTCL cell lines (Fig. 1d). As PTGDS was a kind of secreted protein, we further evaluated the level of serum PTGDS and found that compared with healthy control (*n* = 31), PTCL patients (*n* = 76) displayed lower level of serum PTGDS (*p* < 0.01) (Supplementary Fig. S1D), indicating the reduced secretion of PTGDS protein in PTCL cells.

To investigate the clinical association of PTGDS in PTCL patients, we performed further analysis and found that the high expression of PTGDS in tumor tissue was statistically correlated with ALK^+^ ALCL (*p* = 0.008) and elevated ESR (*p* = 0.004) in PTCL patients (Fig. 1e, Supplementary Fig. S1E). However, there was no significant difference between PTGDS expression and Ann Arbor stage, IPI socre, liver invasion and so on (Supplementary Table 1), which might due to the limited number of samples in different subtypes. Moreover, the high level of serum PTGDS ( > 56.9 ng/mL) was found to be associated with clinical characteristics in PTCL patients, including high IPI score, elevated β2-MG, advanced stage and elevated ESR (Supplementary Fig. S1F, Supplementary Table 3). Furthermore, Kaplan–Meier survival curve showed that PTCL patients with positive expression of PTGDS exhibited shorter overall survival (OS, *p* = 0.001) and progression-free survival (PFS, *p* = 0.003) (Fig. 1f, g). Besides, univariate and multivariate analyses revealed that PTGDS score was independent risk factor for OS and PFS in PTCL patients (Supplementary Tables 5 and 6). These results provided a promising biomarker for the prognostic prediction of PTCL patients, and indicated the potential role of PTGDS in the progression of PTCL.

### Knockdown of PTGDS expression inhibited tumor growth of PTCL cells in vitro and in vivo

**Fig. 2:**
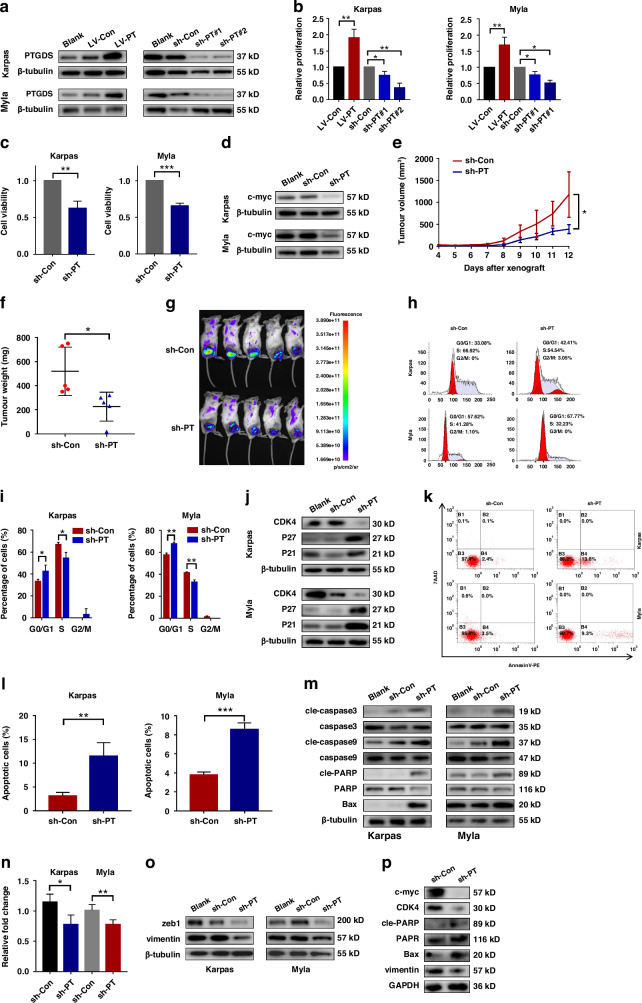
Knockdown of PTGDS expression inhibited tumor growth of PTCL cells in vitro and in vivo. **a** Western blotting was performed to verify the transfection efficiency. **b** Results of CCK8 assay at 48 h showed that PTGDS overexpression increased the proliferation of PTCL cells and PTGDS knockdown decreased it. **c**, **d** PTGDS knockdown inhibited the cell viability and the expression of c-myc in PTCL cells at 36 h. **e**–**g** Compared with sh-Control group, the growth rate, weight, volume and bioluminescence of tumor in mouse model were lower in sh-PTGDS group (*n* = 5 per group). **h**–**j** PTGDS knockdown induced cell cycle arrest at the G0/G1 phase, decreased the expression of CDK4, and increased the expression of CDK inhibitors P21 and P27 in PTCL cells at 36 h. **k**–**m** PTCL cells with PTGDS knockdown displayed higher apoptosis rate, increased expression of Bax and the cleaved forms of caspase-3, caspase-9 and PARP at 36 h. **n**–**o** PTGDS knockdown inhibited cell invasion and the expression of zeb1 and vimentin in PTCL cells at 36 h. **p** PTGDS knockdown regulated the expression of important proteins in tumor tissue from mouse model. Data are shown as the mean ± SD. *p < 0.05; **p < 0.01; ***p < 0.001.

The above findings prompted us to further illuminate the regulatory role of PTGDS in the progression of PTCL, and we performed lentiviral transfection to overexpress and knockdown the expression of PTGDS in PTCL cells. Western blotting was performed to evaluate the transfection efficiency (Fig. 2a). Results of CCK8 assay showed that PTGDS overexpression increased the proliferation of PTCL cells while PTGDS knockdown significantly decreased it (Fig. 2b). Then, sh-PTGDS#2 was chosen for further research based on the efficacy of gene knockdown and the level of proliferation inhibition (Fig. 2a, b). Moreover, rescue experiments revealed that PTGDS overexpression could partly reverse the inhibitory effects of PTGDS knockdown on cell proliferation in PTCL (Supplementary Fig. S2A), confirming the key role of PTGDS in PTCL progression. Besides, PTGDS knockdown was found to suppress the viability of PTCL cells (Fig. 2c) and the expression of c-myc, an essential factor in cell proliferation (Fig. 2d).

To verify the role of targeting PTGDS in vivo, we established a subcutaneous xenograft mouse model using PTCL cells with PTGDS or control knockdown (*n* = 5 per group). Decreased growth rate (Fig. 2e), low tumor volume and weight (Fig. 2f, Supplementary Fig. S2B) were observed in mice bearing PTCL cells with PTGDS knockdown at the end of experiment. Furthermore, results from in-vivo imaging system showed that targeted inhibition of PTGDS could significantly decrease the bioluminescence of tumor cells (Fig. 2g). Overall, the above results indicated that targeting PTGDS could inhibit the proliferation of PTCL cells in vitro and in vivo.

To investigate the role of PTGDS in cell cycle and apoptosis, we performed flow cytometry experiment in PTCL cells with PTGDS or control knockdown. Representative pictures of cell cycle analysis were shown in Fig. 2h, and PTGDS knockdown was observed to induce cell cycle arrest at the G0/G1 phase (*p* < 0.05) (Fig. 2i). Moreover, targeted inhibition of PTGDS could decrease the expression of CDK4, an essential factor in the transformation from G1 phase to S phase, and significantly increase the expression of CDK inhibitors P21 and P27 in PTCL cells (Fig. 2j). Furthermore, PTCL cells with PTGDS knockdown displayed higher apoptosis rate (Fig. 2k, l), increased expression of pro-apoptotic proteins, including Bax (Bcl-2 associated X protein) and the cleaved forms of caspase-3, caspase-9 and PARP (Fig. 2m). Rescue experiments found that the promoting effects of PTGDS knockdown on cell apoptosis could be partly reversed by PTGDS overexpression (Supplementary Fig. S2C), verifying the important role of PTGDS in PTCL progression. Besides, PTGDS knockdown was found to significantly decrease the invasive ability (Fig. 2n), and the expression of zeb1 and vimentin, important positive factors of cell invasion (Fig. 2o) in PTCL cells. Similarly, tumor tissue from mice bearing sh-PTGDS cells displayed decreased expression level of c-myc, CDK4, vimentin and Ki67, and increased expression level of Bax and cleaved PARP (Fig. 2p, Supplementary Fig. S2D). Collectively, these results suggested that PTGDS might play an important role in the development of PTCL through regulating cell viability, proliferation, cell cycle, apoptosis and invasion.

### PTGDS inhibitor AT56 exerted significant anti-tumor effects in PTCL

**Fig. 3:**
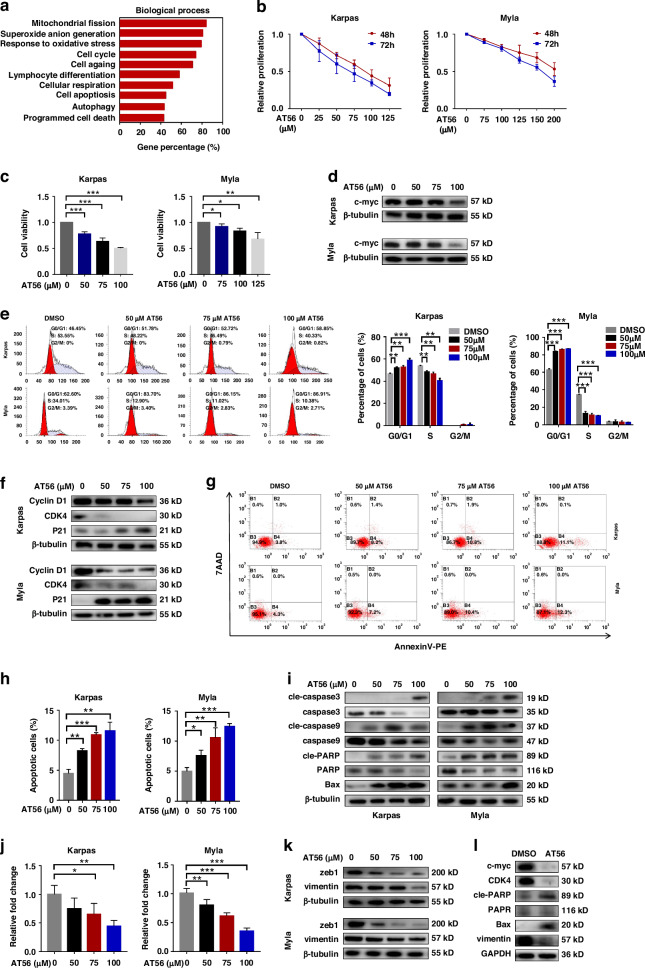
PTGDS inhibitor AT56 exerted significant anti-tumor effects in PTCL. **a** RNA-seq and GO analysis showed the association between PTGDS expression and tumor biology in PTCL. **b**, **c** AT56 inhibited the proliferation and viability of PTCL cells in a dose-dependent manner at 48 h and 72 h. **d** AT56 decreased the expression of c-myc in PTCL cells at 36 h. **e**, **f** PTCL cells treated with AT56 for 36 h displayed dose-dependently cell cycle arrest at G0/G1 phase, the decreased expression of CDK4 and Cyclin D1 and increased expression of P21. **g**–**i** AT56 treatment for 36 h significantly increased the apoptosis rate and the expression of apoptosis-associated proteins in PTCL cells, with concentration dependence. **j**, **k** Inhibited cell invasion and decreased expression of zeb1 and vimentin were found in PTCL cells treated with AT56 for 36 h. **l** AT56 treatment regulated the expression of important proteins in tumor tissue from mouse model. Data are shown as the mean ± SD. *p < 0.05; **p < 0.01; ***p < 0.001.

To illuminate the effects of PTGDS inhibitor AT56 in PTCL development, we performed RNA-seq in PTCL cells treated with AT56 or control, and identified differentially expressed genes (Fold change > 1.5, *p* < 0.05). Gene ontology (GO) analysis based on differentially expressed genes revealed that targeting inhibition of PTGDS was closely associated with tumor biology in PTCL cells, including cell death, cell apoptosis, cell cycle, cell aging, autophagy and so on (Fig. 3a).

Next, we performed further experiments to validate the effects of AT56 in the progression of PTCL. It’s found that AT56 could inhibit the cell proliferation and cell viability of PTCL cells, with concentration dependence (Fig. 3b, c). Besides, AT56 treatment was found to inhibit cell proliferation in sh-Con cells, but not in sh-PTGDS cells (Supplementary Fig. S3A), supporting the specificity of AT56 on PTGDS in PTCL cells. The expression of c-myc was dose-dependently decreased by the treatment with AT56 in PTCL cells (Fig. 3d), indicating the inhibitory effects of AT56 in cell growth in vitro. Moreover, AT56 was found to induce cell cycle arrest at G0/G1 phase in a dose-dependent manner (Fig. 3e). After the treatment with AT56, PTCL cells displayed decreased expression of CDK4 and Cyclin D1 and increased expression of P21 in a dose-dependent manner (Fig. 3f). AT56 treatment could also increase the early apoptotic cell populations of PTCL cells, with concentration dependence (Fig. 3g, h). Obviously, PTCL cells treated with AT56 displayed dose-dependently increased expression of pro-apoptotic proteins, including Bax, and the cleaved forms of caspase-3, caspase-9 and PARP (Fig. 3i). Moreover, apoptosis inhibitor, Z-VAD, was found to partly rescue the anti-proliferation effects of AT56 treatment in PTCL cells, but the difference was not statistically significant (Supplementary Fig. S3B), suggesting that cell apoptosis might be the important but not the major mode of cell death after targeting PTGDS. The dose-dependent reduction of cell invasion was observed in PTCL cells treated with AT56 (Fig. 3j), which was accompanied by the decreased expression of zeb1 and vimentin (Fig. 3k). Similarly, tumor tissue from mice treated with AT56 displayed decreased expression of c-myc, CDK4 and vimentin, and increased expression of cleaved PARP and Bax (Fig. 3l). Taken together, our results indicated that AT56 exerted significant anti-tumor effects in PTCL through regulating cell proliferation, cell viability, cell cycle, cell apoptosis and cell invasion.

### AT56 treatment promoted the ferroptosis process in PTCL cells

**Fig. 4:**
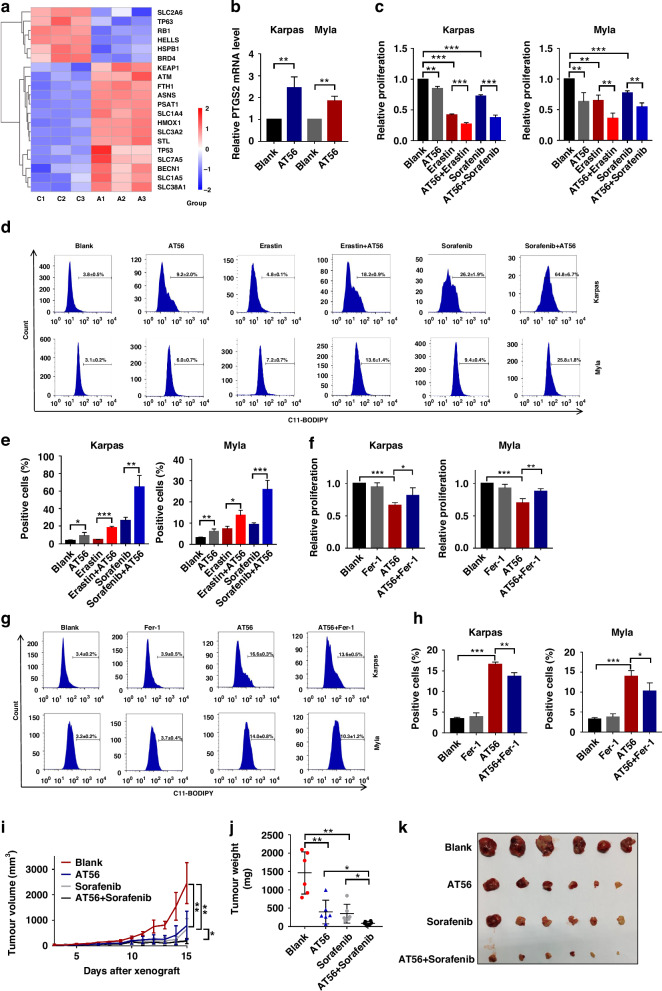
AT56 treatment promoted the ferroptosis process in PTCL cells. **a** Analysis of differentially expressed proteins based on TMT-mass spectrometry showed the potential regulatory role of AT56 treatment on ferroptosis in PTCL. **b** AT56 treatment for 36 h increased the expression of PTGS2 in PTCL cells, classic biomarker of ferroptosis. **c** AT56 enhanced the inhibitory effect of Erastin and Sorafenib on cell proliferation at 36 h. **d**, **e** AT56 treatment for 36 h significantly promoted the Erastin- and Sorafenib-induced accumulation of lipid ROS. **f**–**h** Fer-1 treatment for 36 h partly reversed AT56-induced cell proliferation inhibition and lipid ROS accumulation in PTCL cells. **i**–**k** The combination of AT56 and Sorafenib significantly inhibited tumor growth in PTCL mouse model. Data are shown as the mean ± SD. *p < 0.05; **p < 0.01; ***p < 0.001.

To further explore the regulatory mechanism of AT56 treatment on PTCL progression, we performed TMT-mass spectrometry in PTCL cells treated with AT56 or control. It was found that AT56 treatment could significantly regulate the expression level of ferroptosis-associated proteins in PTCL cells (Fig. 4a). Venn diagram of the overlap between up- and down-regulated ferroptosis-associated molecules in RNA-seq and TMT-mass spectrometry was shown in Supplementary Fig. S4A. KEGG pathway analysis based on the differentially expressed proteins (Fold change > 1.5, *p* < 0.05) in TMT mass spectrometry revealed that AT56 treatment played important role in regulating ferroptosis process in PTCL cells (Supplementary Fig. S4B).

Next, we further determined the regulatory role of AT56 on ferroptosis process in PTCL, and found that AT56 treatment could increase the mRNA expression of PTGS2, the hallmarks of ferroptosis, in PTCL cells (Fig. 4b). As Erastin and Sorafenib have been identified as classic ferroptosis inducers, their inhibitory effects on cell proliferation were significantly enhanced by AT56 treatment in PTCL cells (Fig. 4c). Besides, as Sorafenib functions as both ferroptosis inducer and multi-target kinase inhibitor, we found that the treatment with ferrostatin-1 (Fer-1), a specific inhibitor of ferroptosis, could reverse the proliferation inhibitory effects of Sorafenib, and the combined anti-tumor effects of Sorafenib and AT56 could be reversed by Fer-1 and DFO (Supplementary Fig. S4C), indicating that ferroptosis was the major downstream mechanism. Similarly, results of flow cytometry showed that AT56 treatment promoted the Erastin- and Sorafenib-induced accumulation of lipid ROS, a classic biomarker of ferroptosis (Fig. 4d, e). Notably, the treatment with Fer-1 reversed the inhibition of cell proliferation and the accumulation of lipid ROS in PTCL cells treated with AT56 (Fig. 4f–h), indicating that AT56 treatment might inhibit tumor growth through inducing ferroptosis process in PTCL cells.

To further verify the regulatory effects of AT56 on ferroptosis process in vivo, we constructed PTCL mouse models and found that AT56 treatment could enhance the anti-tumor effects of Sorafenib. Compared with mice received AT56 alone, Sorafenib alone and control, mice received the combination treatment of AT56 and Sorafenib displayed significantly decreased tumor growth rate (Fig. 4I), tumor volume and weight at the end of the experiment (Fig. 4j, k). Moreover, AT56 enhanced the inhibitory role of Sorafenib on the bioluminescence and Ki67 expression of PTCL cells in vivo (Supplementary Fig. S4D–E). Besides, the combination of AT56 and Sorafenib obviously regulated the expression of phenotype-related proteins, including c-myc, CDK4, cleaved PARP, Bax and vimentin (Supplementary Fig. S4F). Altogether, our in-vitro and in-vivo results indicated that AT56 treatment could promote the ferroptosis process and then inhibit tumor growth in PTCL.

### Targeting PTGDS promoted the ferroptosis process in PTCL cells

**Fig. 5:**
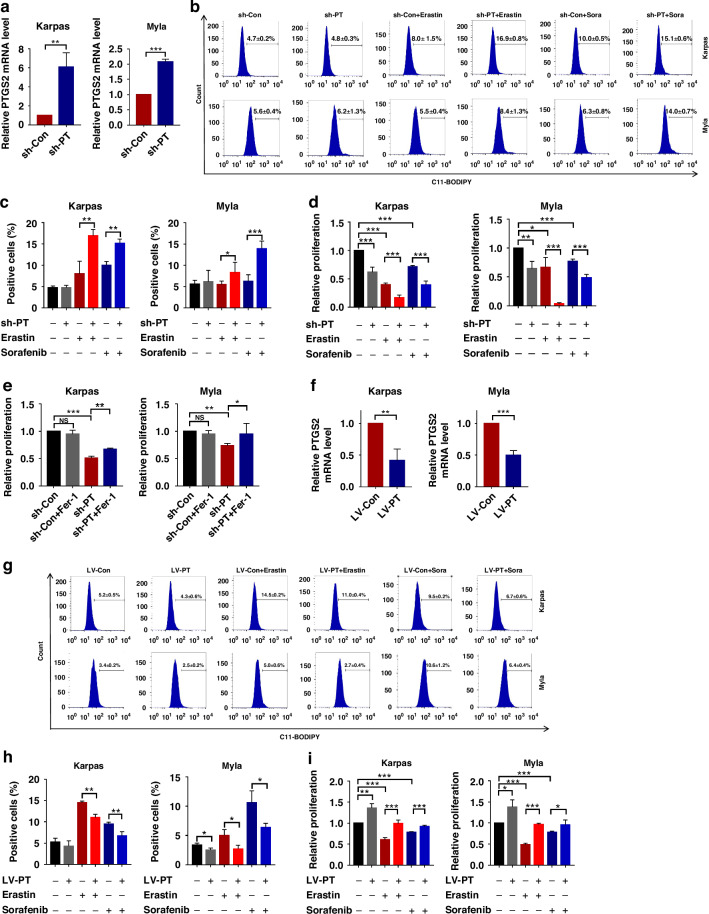
Targeting PTGDS promoted the ferroptosis process in PTCL cells. **a** The expression of PTGS2 mRNA was increased in PTCL cells with PTGDS knockdown at 36 h. **b**, **c** PTGDS knockdown enhanced the promoting effect of Erastin and Sorafenib on lipid ROS accumulation at 36 h. **d** PTGDS knockdown promoted the inhibitory effect of Erastin and Sorafenib on cell proliferation at 36 h. **e** Fer-1 treatment reversed the proliferation inhibition induced by PTGDS knockdown at 36 h. **f** PTGDS overexpression decreased the expression of PTGS2 mRNA at 48 h. **g**–**i** PTGDS overexpression partly reversed the effect of Erastin and Sorafenib on lipid ROS accumulation and proliferation inhibition in PTCL cells at 48 h. Data are shown as the mean ± SD. *p < 0.05; **p < 0.01; ***p < 0.001.

To decipher the regulatory role of targeting PTGDS on ferroptosis in PTCL, we detected ferroptosis-associated phenotypes in PTCL cells with PTGDS knockdown or overexpression. Similarly, PTGDS knockdown increased the expression of PTGS2 mRNA and enhanced the promoting effects of Erastin and Sorafenib on lipid ROS accumulation (Fig. 5a–c). Besides, the inhibitory effects of Erastin and Sorafenib on cell proliferation were significantly enhanced by PTGDS knockdown in PTCL cells (Fig. 5d). We also found that the treatment with Fer-1 and DFO could reverse the combined anti-tumor effects of Sorafenib and PTGDS knockdown in PTCL cells (Supplementary Fig. S5), indicating that ferroptosis played important role in the inhibitory effects. Notably, Fer-1 treatment could partly reverse the proliferation inhibition induced by PTGDS knockdown in PTCL cells (Fig. 5e).

Furthermore, PTCL cells with PTGDS overexpression displayed decreased expression of PTGS2 mRNA (Fig. 5f). PTGDS overexpression was found to reverse the regulatory effects of Erastin and Sorafenib on the accumulation of lipid ROS (Fig. 5g, h) and cell proliferation (Fig. 5i) in PTCL cells. Taken together, the above results indicated that targeting PTGDS might inhibit the development of PTCL through promoting ferroptosis process.

### Targeting PTGDS promoted ferroptosis process through regulating iron metabolism in PTCL cells

**Fig. 6:**
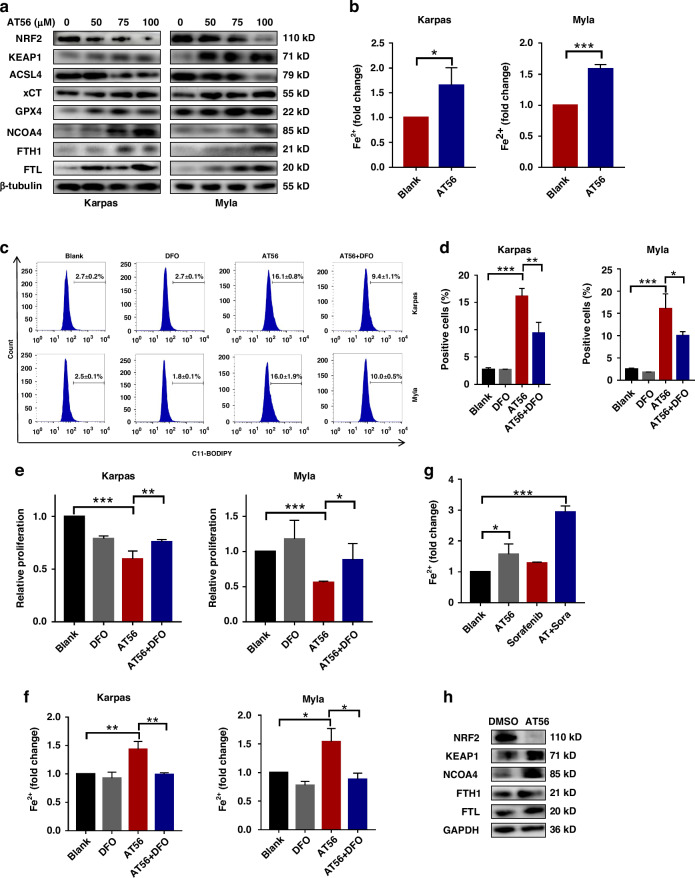
Targeting PTGDS promoted ferroptosis process through regulating iron metabolism in PTCL cells. **a** Western blotting results showed the expression level of ferroptosis-associated proteins in PTCL cells treated with AT56 at 36 h. **b** AT56 treatment for 36 h increased the level of Fe^2+^ in PTCL cells. **c**, **d** The increased accumulation of lipid ROS caused by AT56 was partly reversed by DFO in PTCL cells at 36 h. **e** DFO treatment for 48 h partly reversed the proliferation inhibitory role of AT56 in PTCL cells. **f** The AT56-induced accumulation of Fe^2+^ was reversed by DFO treatment for 36 h in PTCL cells. **g** The level of Fe^2+^ was higher in tumor tissue from mice receiving AT56 and Sorafenib. **h** Western blotting results showed the expression level of ferroptosis-associated proteins in tumor tissue from mouse model. Data are shown as the mean ± SD. *p < 0.05; **p < 0.01; ***p < 0.001.

The major mechanisms of ferroptosis include iron accumulation, ROS production and lipid peroxidation, which are regulated by a complex regulatory network [4, 8]. As targeting PTGDS was found to promote ferroptosis process in PTCL cells, we further performed experiments to explore underlying molecular mechanism. Results of western blotting showed that AT56 treatment decreased the expression of ferroptosis-associated transcription factor NRF2, increased the expression of KEAP1, the negative regulator of NRF2, in PTCL cells in a dose-dependent manner (Fig. 6a). Similar results were found in PTCL cells with PTGDS knockdown (Supplementary Fig. S6A), indicating the enhanced ferroptosis process. Mechanically, the expression of ACSL4, the key factor in lipid metabolism, was decreased in PTCL cells with AT56 treatment and PTGDS knockdown (Fig. 6a, Supplementary Fig. S6A), indicating that targeting PTGDS promoted ferroptosis not through inducing lipid metabolism. Besides, both AT56 treatment and PTGDS knockdown increased the expression of key antioxidant regulators, xCT and GPX4 (Fig. 6a), indicating the compensatory activation of antioxidant system in ferroptosis process.

Furthermore, PTCL cells with AT56 treatment and PTGDS knockdown displayed significantly increased expression of NCOA4, FTH1 and FTL, which were important factors involved in iron metabolism [4]. Both AT56 treatment and PTGDS knockdown could increase the accumulation of bioactive Fe^2+^ in PTCL cells (Fig. 6b, Supplementary Fig. S6B), indicating that targeting PTGDS might promote ferroptosis process through regulating iron metabolism in PTCL.

Moreover, iron chelator DFO was found to reverse the proliferation inhibitory and lipid ROS promoting effects of AT56 treatment in PTCL cells (Fig. 6c–e). Similarly, PTCL cells with PTGDS knockdown displayed higher level of cell proliferation after the treatment with DFO (Supplementary Fig. S6C). The increased level of bioactive Fe^2+^ caused by AT56 treatment and PTGDS knockdown was reversed by DFO treatment in PTCL cells (Fig. 6f, Supplementary Fig. S6D), indicating the key role of iron metabolism in ferroptosis induced by targeting PTGDS in PTCL.

The regulatory role of PTGDS on iron metabolism in PTCL was further confirmed in vivo using a xenograft mouse model. Compared with mice receiving control treatment, tumor tissue from mice with AT56 treatment and PTGDS knockdown displayed increased level of Fe^2+^ (Fig. 6g, Supplementary Fig. S6E). Moreover, targeting PTGDS increased the expression of KEAP1, NCOA4, FTH1, FTL, and decreased the expression of NRF2 (Fig. 6h, Supplementary Fig. S6F) in vivo, which were consistent with the in-vitro results. Taken together, above results indicated that targeting PTGDS might promote ferroptosis process and tumor development through regulating iron metabolism in PTCL.

### Targeting PTGDS promoted iron accumulation and ferroptosis partly through inducing ferritin autophagy in PTCL

**Fig. 7:**
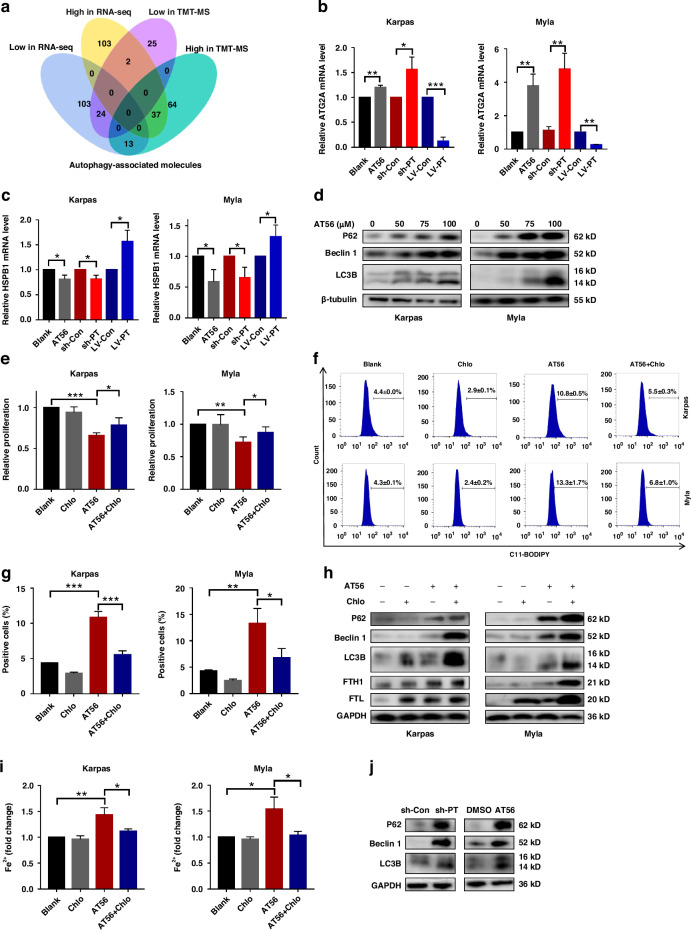
Targeting PTGDS promoted iron accumulation and ferroptosis through inducing ferritin autophagy in PTCL. **a** Venn diagram showed the overlap between up- and down-regulated autophagy-associated molecules in RNA-seq and TMT-mass spectrometry. There were 61 (low, *n* = 24; high, *n* = 37) molecules with consistent expression change trend in RNA-seq and TMT-mass spectrometry. **b**, **c** The expression level of ATG2A and HSPB1 was regulated by AT56 treatment, PTGDS knockdown and overexpression at 36 h. **d** AT56 treatment for 36 h increased the expression of P62, Beclin 1 and LC3B in PTCL cells. **e** Chloroquine treatment for 48 h partly reversed the inhibitory effects of AT56 on the proliferation of PTCL cells. **f**, **g** The promoting role of AT56 on lipid ROS accumulation was partly reversed by chloroquine treatment for 36 h. **h** Chloroquine enhanced the role of AT56 on the expression of autophagy- and iron metabolism-associated proteins at 36 h. **i** The accumulation of Fe^2+^ caused by AT56 was reversed by chloroquine treatment for 36 h in PTCL cells. **j** Western blotting results showed the expression of autophagy-associated proteins in mouse model. Data are shown as the mean ± SD. *p < 0.05; **p < 0.01; ***p < 0.001.

To further explore the underlying mechanism of PTGDS on ferroptosis and iron metabolism in PTCL, we deeply analyzed the data from TMT-mass spectrometry and RNA-seq, and revealed the potential regulatory role of PTGDS on the expression of autophagy-associated molecules (Fig. 7a). As autophagy has been demonstrated to play key roles in the initiation of ferroptosis [8], we performed further experiments to illuminate the regulatory relationship between autophagy and ferroptosis when targeting PTGDS in PTCL cells.

Firstly, to elucidate the regulatory of targeting PTGDS on autophagy process, we found that both AT56 treatment and PTGDS knockdown could increase the mRNA level of ATG2A, which was recognized to promote the formation of autophagosomes, while PTGDS overexpression decreased it (Fig. 7b). Meanwhile, the mRNA expression of HSPB1, a negative regulator of autophagy, was decreased by AT56 treatment and PTGDS knockdown, and increased by PTGDS overexpression in PTCL cells (Fig. 7c). Moreover, AT56 treatment and PTGDS knockdown increased the expression of key autophagy regulators, including P62, Beclin 1 and LC3B in PTCL cells (Fig. 7d, Supplementary Fig. S7A), indicating the increased formation of autophagosome and the activation of autophagy process.

Next, we performed experiments to decipher whether targeting PTGDS promoted ferroptosis through regulating autophagy in PTCL cells. Chloroquine, a classic autophagy inhibitor through suppressing the degradation in lysosomes, could partly reverse the inhibitory effects of AT56 treatment and PTGDS knockdown on the proliferation of PTCL cells (Fig. 7e, Supplementary Fig. S7B). Besides, the promoting role of AT56 on lipid ROS accumulation was partly reversed by chloroquine (Fig. 7f, g). Chloroquine treatment could also enhance the promoting role of AT56 treatment in the expression of autophagy-associated proteins (P62, Beclin 1 and LC3B) and iron metabolism-associated proteins (FTH1 and FTL) (Fig. 7h). Furthermore, the accumulation of Fe^2+^ caused by AT56 treatment and PTGDS knockdown was reversed by chloroquine in PTCL cells (Fig. 7i, Supplementary Fig. S7C). Besides, the regulatory role of targeting PTGDS on the expression of autophagy-associated proteins was confirmed in tumor tissue from mouse models (Fig. 7j). Overall, all the above results indicated that targeting PTGDS promoted iron accumulation and ferroptosis through inducing ferritin autophagy in PTCL

### PTGDS interacted with HMOX1 to regulate iron metabolism and ferroptosis process in PTCL

**Fig. 8:**
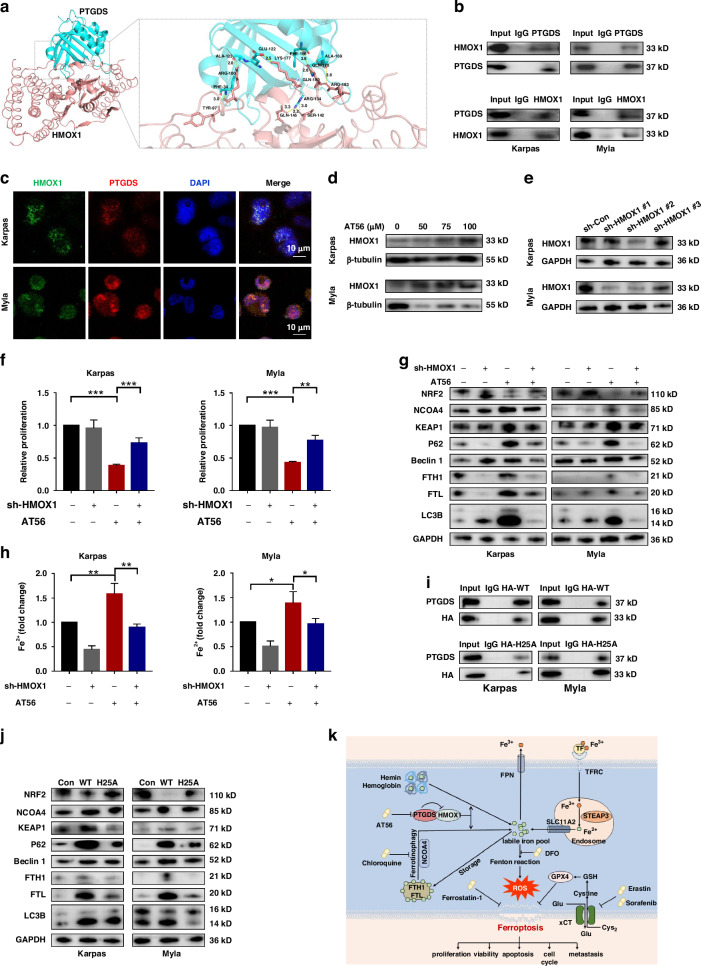
PTGDS interacted with HMOX1 to regulate iron metabolism and ferroptosis process in PTCL. **a** Molecular docking pattern diagrams of PTGDS protein (blue) and HMOX1 protein (red). Confidence score = 0.8. **b**, **c** The colocalization and interaction between PTGDS and HMOX1 were verified in PTCL cells through confocal immunofluorescent and Co-IP. Bar = 10 μm. **d** AT56 treatment for 36 h increased the expression level of HMOX1 in PTCL cells. **e** Western blotting results verified the transfection efficiency. **f** HMOX1 knockdown reversed the inhibitory role of AT56 on cell proliferation at 48 h. **g**, **h** The regulatory role of AT56 on the expression of ferroptosis- and autophagy-associated proteins, and Fe^2+^ accumulation was reversed by HMOX1 knockdown at 36 h. **i** Co-IP assay showed the interaction between PTGDS protein and HMOX1 protein with wild type and H25A mutation. **j** H25A mutation reversed the regulatory role of HMOX1 on the expression of ferroptosis- and autophagy-associated proteins. **k** Mechanism diagram summarized that PTGDS promotes the development of PTCL through regulating HMOX1-mediated iron metabolism and ferroptosis process, and targeting PTGDS could exert synergistic anti-tumor effects with ferroptosis inducers Erastin and Sorafenib in PTCL. Data are shown as the mean ± SD. *p < 0.05; **p < 0.01; ***p < 0.001.

Molecular docking and interaction relationship analysis of PTGDS and differentially expressed ferroptosis-associated molecules in TMT-mass spectrometry and RNA-seq was performed to elucidate the precise mechanism through which targeting PTGDS promotes ferroptosis process in PTCL (Fig. 8a, Supplementary Fig. S8A). Among them, HMOX1 protein, the rate-limiting enzyme in the degradation of heme to release free iron, was found to possess potential interactions with PTGDS protein (Fig. 8a), and HMOX1 has been demonstrated to play key role in the process of ferroptosis and autophagy [22, 23]. Furthermore, results of confocal immunofluorescent and Co-IP verified the colocalization and interaction between PTGDS and HMOX1 in PTCL cells (Fig. 8b, c). Both AT56 treatment and PTGDS knockdown could increase the expression level of HMOX1 in PTCL cells (Fig. 8d, Supplementary Fig. S8B).

To decipher whether HMOX1 was involved in the regulatory role of PTGDS on ferroptosis in PTCL, we performed lentiviral transfection to knockdown the expression of HMOX1 in PTCL cells, and the transfection efficiency was validated by western blotting (Fig. 8e). HMOX1 knockdown could reverse the inhibitory role of AT56 treatment on the proliferation of PTCL cells (Fig. 8f). Moreover, the regulatory role of AT56 on the expression of ferroptosis-associated and autophagy-associated proteins, and the accumulation of Fe^2+^ was reversed by HMOX1 knockdown in PTCL cells (Fig. 8g, h), indicating that HMOX1 played a key role in the positive effect of targeting PTGDS on iron metabolism and ferroptosis process in PTCL.

As cytoplasmic protein HMOX1 is mainly anchored on endoplasmic reticulum, the lack of C-terminal transmembrane segment could induce the translocation of HMOX1 protein to nucleus, which enhances the growth and invasiveness of tumor cells [24–26]. Our results showed that the treatment with AT56 had no effect on the cytosolic and nuclear distribution of HMOX1 protein in PTCL cells (Supplementary Fig. S8C). Furthermore, previous research revealed that HMOX1 with H25A mutation lost the catalytic activity to degrade heme and release free iron [27]. In our study, H25A mutation had no effect on the interaction between HMOX1 proteins and PTGDS proteins (Fig. 8i). Besides, wild type HMOX1 could regulate the expression of ferroptosis-associated and autophagy-associated molecules in PTCL cells (Fig. 8j), suggesting the promoting role of HMOX1 in the ferroptosis and autophagy process in PTCL. However, HMOX1 with H25A mutation displayed no such effect (Fig. 8j), indicating that targeting PTGDS promoted the process of ferroptosis and autophagy through enhancing the catalytic activity of HMOX1. Collectively, these results revealed that the interaction of PTGDS and HMOX1 could promote the progression of PTCL through inhibiting ferroptosis process, which was mediated by HMOX1-mediated heme degradation and ferritin autophagy (Fig. 8k).

## Discussion

In this study, our observations firstly revealed that the high expression of PTGDS was correlated with worse prognosis in PTCL patients. Targeting PTGDS displayed potential therapeutic efficacy through inhibiting cell viability, proliferation, invasion and inducing apoptosis and cell cycle arrest in vitro and in vivo. Moreover, targeting PTGDS could promote ferroptosis process and enhance the sensitivity of PTCL cells to ferroptosis inducers Sorafenib. Mechanically, targeting PTGDS could inhibit the progression of PTCL through inducing ferroptosis process and iron metabolism, which was mediated by HMOX1-mediated heme degradation and ferritin autophagy. The specific gene site of HMOX1 corresponding to its function while targeting PTGDS was verified by point mutation in PTCL. These inspiring findings provide novel therapeutic target and strategies to improve the efficacy of ferroptosis-targeted therapy in the treatment of PTCL patients.

As a key enzyme in prostaglandin metabolism, PTGDS has been identified as potential prognostic biomarker and important regulator in tumor development [28]. Previous investigations reported that PTGDS was highly expressed in high‑grade serous ovarian carcinoma [29], the endothelial cells of melanoma and oral squamous cell carcinoma [30]. The decreased expression of PTGDS was associated with TNM stage and worse prognosis in tumor patients [31–34]. Moreover, previous studies have demonstrated that PTGDS served as a tumor suppressing gene in solid tumors [30, 35]. Analysis based on genome-wide CRISPR-Cas9 knock-out screening showed that PTGDS inhibited the invasion and metastasis of prostate cancer [36]. Moreover, AT56, the specific PTGDS inhibitor [37], reversed the regulatory role of PTGDS on neuroprotective properties [38], and inhibited the cell proliferation in prostate fibroblasts and epithelial cells [39], indicating its therapeutic value. In our study, the high expression of PTGDS protein was correlated with worse prognosis in PTCL patients, and targeting PTGDS by gene knockdown and AT56 treatment could suppress tumor growth in vitro and in vivo through regulating cell proliferation, cell cycle, cell apoptosis and invasion. The discrepancy of the regulatory role of PTGDS in different tumors might result from the diverse downstream regulatory networks, the high heterogeneity of tumor cells, and the complexity of tumor microenvironment. Our results indicate that PTGDS has a potential capability for prognostic prediction, and targeting PTGDS might serve as promising therapeutic strategy in PTCL treatment. Further research with longer follow-up duration and larger number of sample populations may be needed in future to evaluate the clinical application value of PTGDS in PTCL patients.

PTGDS has been found to serve as critical regulator in tumor development by regulating multiple signaling pathways and complex regulatory networks. As the key metabolic enzyme in PGD2 production, PTGDS knockdown reduced the level of PGD2 in gastric cancer stem cells, and promoted the viability, invasion, and stemness of tumor cells through binding to PGD2 receptor [14, 40]. Moreover, in-vivo study found that PTGDS deficiency promoted the growth of melanoma through accelerating vascular hyperpermeability, angiogenesis, and endothelial-to-mesenchymal transition, which could be reversed by PGD2 receptor agonist [30]. Besides, PTGDS-PGD2 could suppress tumor growth by inhibiting the phosphorylation and nuclear expression of STAT3 in gastric cancer [14], reducing the expression of transcription factor TWIST2 in breast cancer [35], and activating PPARγ ligand-binding domain and the peroxisome proliferator response element reporter systems in prostate tumor [13]. PTGDS overexpression decreased the invasiveness of lung adenocarcinoma cells via the regulation of MAPK signal pathway [12]. Furthermore, we have reported that PTGDS inhibition led to reduced expression of MYH9, and then declined activation of the Wnt-β-catenin-STAT3 pathway through influencing the ubiquitination and degradation of GSK3-β in DLBCL [11]. Here, RNA-sequencing and quantitative TMT proteomics revealed the potential regulatory role of PTGDS on ferroptosis in PTCL cells, which was verified in further mechanism study.

Ferroptosis, a recently identified form of regulated cell death, has been characterized by iron accumulation, ROS production and lipid peroxidation. Recent studies have revealed that ferroptosis plays key roles in the development of several tumors [5, 10, 41], including lymphoma [42]. The combination of zanubrutinib and navitoclax could promote the ferroptosis process and inhibit cell growth in double-hit lymphoma [43], and artesunate displayed antitumor effects through inducing ferroptosis in the mouse models of burkitt’s lymphoma [44]. In-vitro and in-vivo experiments found that the increased level of ferroptosis enhanced by iron oxide nanoparticles and drug treatment, such as artesunate and imidazole ketone Erastin, could significantly inhibit the growth of DLBCL through inducing intracellular iron accumulation [45], lipid peroxidation and ferritinophagy [46], and impairing the STAT3 signaling pathway [47] in lymphoma. Here, our results revealed that targeting PTGDS inhibited the progression of PTCL through inducing iron accumulation and ferroptosis process both in vitro and in vivo. Therefore, above results provide a novel theoretical basis for the mechanistic investigation of PTCL development, and offer molecular targets and intervention strategies for the individual treatment of PTCL patients. In our study, although there was similarity between TMT-mass spectrometry and RNA-sequencing after AT56 treatment, only the differentially expressed molecules in TMT-mass spectrometry was enriched in ferroptosis, and the discrepancy might result from diverse post-translational modifications, which needs to be further explored. Deeper insight into the regulatory network and molecular mechanisms involved in the ferroptosis process in PTCL remains to be fully elucidated, and more therapeutic targets are still to be identified to improve the efficacy of ferroptosis-targeted therapy in PTCL.

HMOX1 is an inducible rate-limiting enzyme that degrades heme into free iron, carbon monoxide and biliverdin [24], and H25A site mutation could destruct its role in iron metabolism [27, 48]. Intracellular HMOX1 is mainly anchored on endoplasmic reticulum to catalyze heme degradation [49], and the lack of C-terminal transmembrane segment results in its nuclear translocation [25, 26], which exerts regulatory role in the activity of transcription factors. Recently, HMOX1 has been revealed to play key roles in the process of ferroptosis and disease progression, especially in tumor development [50, 51], which provides novel potential molecular targets for disease treatment. Besides, the overexpression and over-activation of HMOX1 has been involved in triggering autophagy in tumor development [23, 52, 53]. In our study, HMOX1 was found to interact with PTGDS and mediate the tumor promoting role of PTGDS in PTCL through promoting iron metabolism and autophagy-dependent ferroptosis. The pool of bioactive Fe^2+^ is in a dynamic equilibrium, which is mainly controlled by the entrance of extracellular iron, the production from HMOX1-meidated heme degradation, the storage as ferritin, and the release of Fe^2+^ mediated by ferritin autophagy. We found that PTGDS inhibition promoted the production, storage and release of Fe^2+^ in PTCL cells, and the increased expression level of ferritin indicated that the storage of Fe^2+^ was faster than its release. Moreover, although transcription factor NRF2, the positive regulator of HMOX1 expression [54, 55], was down-regulated by PTGDS inhibition, the expression of HMOX1 protein was increased in PTCL cells, and the interaction between PTGDS protein and HMOX1 protein might have influence on the stability of HMOX1 protein in PTCL cells, which needs to be further explored. Besides, targeting PTGDS had no effect on the cytosolic and nuclear distribution of HMOX1 protein in PTCL cells. Moreover, analysis based on H25A site mutation showed that the regulatory role of HMOX1 in ferroptosis process and PTCL development mainly depended on its catalytic function on iron metabolism. Further investigations on the detailed molecular mechanisms involved in HMOX1 in PTCL are still needed.

Sorafenib, a kind of multi-targeted tyrosine kinase inhibitor, could promote ferroptosis process through inhibiting the activity of system Xc^-^. Sorafenib and its derivatives have become standard first-line treatment regimens for hepatocellular carcinoma patients [56, 57], and patients with desmoid tumors and renal cell carcinoma also benefited from the treatment of Sorafenib [58, 59]. As the low response rate [60], adverse effects [61] and drug resistance [62] limit its clinical application, the combination of Sorafenib with other antitumor therapy, such as immunotherapeutic agent R848 [63], PARP inhibitor olaparib [64], and CDK12 inhibitor THZ531 [65], was found to enhance its therapeutic efficiency through reprogramming tumor immune microenvironment, facilitating vascular normalization, and inducing apoptosis or senescence. Furthermore, Sorafenib displayed superior antitumor efficacy in FLT3-ITD acute myeloid leukemia patients [66], especially undergoing allogeneic hematopoietic stem-cell transplantation [67, 68]. Results from clinical trial showed that Sorafenib had a clinical activity in the treatment of lymphoma [69], but with low single agent activity [70] and high adverse events [71]. The combination of Sorafenib with other anti-lymphoma drugs, such as decitabine [72], vorinostat [73] and bortezomib [74], showed a synergistic antitumor effect in lymphoma treatment. Our present study found that the addition of AT56 significantly enhanced the antitumor effect of Sorafenib in PTCL cells and xenograft mouse model through promoting ferroptosis process, which provided promising combination therapy for PTCL patients. Further studies are warranted to investigate the efficiency and mechanism of combination therapy based on Sorafenib in PTCL patients.

However, there are still some limitations in our study. Firstly, the prognostic analysis of PTGDS was restricted by its retrospective nature and the small sample size. Further prospective and large-scale multicenter research with larger sample sizes are needed to verify the clinical value of PTGDS in PTCL patients. Secondly, mice models with cell line-derived xenograft were used in the study, and patient-derived xenograft (PDX) models could better simulate the growth of tumors in patients’ body. Lastly, further preclinical studies about AT56 in PDX mice model are needed to promote its clinical application in PTCL treatment, including pharmacokinetic and pharmacodynamic studies for searching its optimal dose, acute and long-term toxicity test, allergic and hemolytic test, mutagenic and teratogenic test, reproductive toxicity test and so on.

Collectively, our observations firstly revealed the high expression and prognostic role of PTGDS in PTCL patients, and targeting PTGDS displayed excellent anti-lymphoma effects and enhanced the drug sensitivity of PTCL cells to Sorafenib in vitro and in vivo. Mechanically, targeting PTGDS increased the level of iron and induced ferroptosis in PTCL through promoting HMOX1-mediated heme catabolism and ferritin autophagy process. Point mutation verified the specific gene site of HMOX1 corresponding to its function while targeting PTGDS. Overall, our inspiring findings demonstrated that targeting PTGDS promoted the ferroptosis in PTCL through regulating HMOX1-mediated iron metabolism, and provided novel prognostic biomarkers, molecular targets and combination regimen for PTCL treatment.

## Supplementary information

Supplementary Table 1

Supplementary Table 2

Supplementary Table 3

Supplementary Table 4

Supplementary Table 5

Supplementary Table 6

Supplementary materials and figures

## Data Availability

The datasets used and/or analyzed during the current study are available from the corresponding authors on reasonable request.
