# Supplementary material for: Targeting PTGDS Promotes ferroptosis in peripheral T cell lymphoma through regulating HMOX1-mediated iron metabolism
Source: Br J Cancer. 2024 Dec 20;132(4):384–400. doi: 10.1038/s41416-024-02919-w (PMC11833084; doi:10.1038/s41416-024-02919-w)
Supplement: Supplementary file 1 — Supplementary Table 1 [file 41416_2024_2919_MOESM1_ESM.docx]

**Supplemental Table 1.** Clinical characteristics based on PTGDS expression in PTCL patients.

| Characteristics | No. of  patients | Negative PTGDS  (number) | Positive PTGDS  (number) | P value |
| --- | --- | --- | --- | --- |
| **Age(years)** |  |  |  |  |
| <60 | 117 | 56 | 61 | 0.369 |
| ≥60 | 42 | 24 | 18 |  |
| **Gender** |  |  |  |  |
| Male | 106 | 54 | 52 | 0.867 |
| Female | 53 | 26 | 27 |  |
| **Ann Arbor Stage** |  |  |  |  |
| Ⅰ/Ⅱ | 50 | 28 | 22 | 0.393 |
| Ⅲ/Ⅳ | 107 | 51 | 56 |  |
| **IPI score > 3** |  |  |  |  |
| No | 77 | 43 | 34 | 0.181 |
| Yes | 66 | 29 | 37 |  |
| **B symptom** |  |  |  |  |
| Yes | 81 | 44 | 37 | 0.340 |
| No | 76 | 35 | 41 |  |
| **Decreased ALB** |  |  |  |  |
| No | 39 | 16 | 23 | 0.200 |
| Yes | 118 | 63 | 55 |  |
| **Elevated ESR** |  |  |  |  |
| No | 26 | 18 | 8 | 0.060 |
| Yes | 59 | 27 | 32 |  |
| **Liver invasion** |  |  |  |  |
| No | 114 | 54 | 60 | 0.283 |
| Yes | 43 | 25 | 18 |  |
| **Spleen invasion** |  |  |  |  |
| No | 115 | 55 | 60 | 0.368 |
| Yes | 42 | 24 | 18 |  |
| **Marrow invasion** |  |  |  |  |
| No | 138 | 72 | 66 | 0.325 |
| Yes | 18 | 7 | 11 |  |
| **Central invasion** |  |  |  |  |
| No | 148 | 74 | 74 | 0.712 |
| Yes | 7 | 4 | 3 |  |
| **EB virus infection** |  |  |  |  |
| No | 55 | 23 | 32 | 0.148 |
| Yes | 68 | 38 | 30 |  |
| **Therapeutic Efficacy** |  |  |  |  |
| CR+PR | 26 | 12 | 14 | 1.000 |
| SD+PD | 68 | 33 | 35 |  |

Abbreviations: IPI, international prognostic index; ALB, albumin; ESR, erythrocyte sedimentation rate; EB, Epstein-Barr; CR, complete remission; PR, partial remission; SD, stable disease; PD, progressive disease.
