# Supplementary material for: Targeting PTGDS Promotes ferroptosis in peripheral T cell lymphoma through regulating HMOX1-mediated iron metabolism
Source: Br J Cancer. 2024 Dec 20;132(4):384–400. doi: 10.1038/s41416-024-02919-w (PMC11833084; doi:10.1038/s41416-024-02919-w)
Supplement: Supplementary file 3 — Supplementary Table 3 [file 41416_2024_2919_MOESM3_ESM.docx]

**Supplemental Table 3.** Clinical characteristics based on serum PTGDS concentration in PTCL patients.

| Characteristics | No. of  patients | Low PTGDS  n (%) | High PTGDS  n (%) | P value |
| --- | --- | --- | --- | --- |
| **Age(years)** |  |  |  |  |
| <60 | 50 | 31（41%） | 19（25%） | 0.101 |
| ≥60 | 26 | 11（14%） | 15（20%） |  |
| **Gender** |  |  |  |  |
| Male | 55 | 30（39%） | 25（33%） | 0.839 |
| Female | 21 | 12（16%） | 9（12%） |  |
| **Ann Arbor Stage** |  |  |  |  |
| Ⅰ/Ⅱ | 23 | 17（22%） | 6（8%） | **0.031** |
| Ⅲ/Ⅳ | 53 | 25（33%） | 28（37%） |  |
| **IPI score > 3** |  |  |  |  |
| No | 48 | 31（41%） | 17（22%） | **0.032** |
| Yes | 28 | 11（15%） | 17（22%） |  |
| **B symptom** |  |  |  |  |
| Yes | 34 | 16（21%） | 18（24%） | 0.196 |
| No | 42 | 26（34%） | 16（21%） |  |
| **Elevated ESR** |  |  |  |  |
| Yes | 22 | 9（22%） | 13（33%） | **0.048** |
| No | 18 | 13（33%） | 5（12%） |  |
| **Liver invasion** |  |  |  |  |
| Yes | 33 | 18（24%） | 15（20%） | 0.912 |
| No | 43 | 24（31%） | 19（25%） |  |
| **Spleen invasion** |  |  |  |  |
| Yes | 22 | 11（14%） | 11（14%） | 0.556 |
| No | 54 | 31（41%） | 23（31%） |  |
| **Marrow invasion** |  |  |  |  |
| Yes | 15 | 8（11%） | 7（9%） | 0.867 |
| No | 61 | 34（45%） | 27（35%） |  |
| **Central invasion** |  |  |  |  |
| Yes | 4 | 2（3%） | 2（3%） | 0.609 |
| No | 72 | 40（52%） | 32（42%） |  |
| **EB virus infection** |  |  |  |  |
| Yes | 37 | 20（26%） | 17（23%） | 0.916 |
| No | 38 | 21（58%） | 17（23%） |  |
| **Therapeutic Efficacy** |  |  |  |  |
| CR+PR | 18 | 9（12%） | 9（12%） | 0.596 |
| SD+PD | 56 | 32（43%） | 24（33%） |  |

Abbreviations: IPI, international prognostic index; ESR, erythrocyte sedimentation rate; EB, Epstein-Barr; CR, complete remission; PR, partial remission; SD, stable disease; PD, progressive disease.
