# Supplementary material for: Targeting PTGDS Promotes ferroptosis in peripheral T cell lymphoma through regulating HMOX1-mediated iron metabolism
Source: Br J Cancer. 2024 Dec 20;132(4):384–400. doi: 10.1038/s41416-024-02919-w (PMC11833084; doi:10.1038/s41416-024-02919-w)
Supplement: Supplementary file 5 — Supplementary Table 5 [file 41416_2024_2919_MOESM5_ESM.docx]

**Supplemental Table 5. Univariate and multivariate analyses of overall survival in PTCL patients.**

|  | **Univariate analysis** | | **Multivariate analysis** | |
| --- | --- | --- | --- | --- |
| **Characteristics** | **HR[95%CI]** | **P value** | **HR[95%CI]** | **P value** |
| **Age(>60)** | 1.836[1.030-3.273] | **0.039** | 1.584[0.835-3.007] | 0.159 |
| **Gender (Male)** | 0.741[0.424-1.294] | 0.292 |  |  |
| **Ann Arbor Stage (Ⅲ/Ⅳ)** | 3.883[1.658-9.095] | **0.002** | 1.638[0.609-4.407] | 0.329 |
| **IPI score( > 3)** | 3.596[1.968-6.574] | **<0.001** | 1.853[0.877-3.914] | 0.106 |
| **B symptom** | 1.972[1.136-3.421] | **0.016** | 1.324[0.720-2.434] | 0.367 |
| **Elevated ESR** | 1.209[0.508-2.874] | 0.668 |  |  |
| **Decreased ALB** | 1.686[0.846-3.360] | 0.137 |  |  |
| **Liver invasion** | 1.850[1.060-3.230] | **0.030** | 1.492[0.773-2.878] | 0.233 |
| **Spleen invasion** | 2.275[1.310-3.949] | **0.004** | 1.509[0.822-2.769] | 0.184 |
| **Marrow invasion** | 1.235[0.557-2.739] | 0.603 |  |  |
| **Central invasion** | 2.491[0.989-6.272] | 0.053 |  |  |
| **EB virus infection** | 0.585[0.331-1.033] | 0.065 |  |  |
| **PTGDS positive** | 2.580[1.445-4.609] | **0.001** | 2.718[1.478-5.001] | **0.001** |

Abbreviations: IPI, international prognostic index; ESR, erythrocyte sedimentation rate; ALB, albumin; EB, Epstein-Barr; HR, hazard ratio.
