# Supplementary material for: Targeting PTGDS Promotes ferroptosis in peripheral T cell lymphoma through regulating HMOX1-mediated iron metabolism
Source: Br J Cancer. 2024 Dec 20;132(4):384–400. doi: 10.1038/s41416-024-02919-w (PMC11833084; doi:10.1038/s41416-024-02919-w)
Supplement: Supplementary file 6 — Supplementary Table 6 [file 41416_2024_2919_MOESM6_ESM.docx]

**Supplemental Table 6. Univariate and multivariate analyses of progression-free survival in PTCL patients.**

|  | **Univariate analysis** | | **Multivariate analysis** | |
| --- | --- | --- | --- | --- |
| **Characteristics** | **HR[95%CI]** | **P value** | **HR[95%CI]** | **P value** |
| **Age(>60)** | 1.800[1.015-3.193] | **0.044** | 2.255[1.070-4.751] | **0.033** |
| **Gender (Male)** | 0.777[0.450-1.340] | 0.364 |  |  |
| **Ann Arbor Stage (Ⅲ/Ⅳ)** | 3.482[1.573-7.708] | **0.002** | 1.782[0.611-5.192] | 0.290 |
| **IPI score( > 3)** | 3.238[1.816-5.774] | **<0.001** | 1.550[0.726-3.310] | 0.257 |
| **B symptom** | 1.785[1.044-3.053] | **0.034** | 0.705[0.366-1.357] | 0.295 |
| **Elevated ESR** | 1.027[0.452-2.338] | 0.948 |  |  |
| **Decreased ALB** | 1.624[0.838-3.148] | 0.151 |  |  |
| **Liver invasion** | 1.850[1.066-3.211] | **0.029** | 3.730[1.625-8.560] | **0.002** |
| **Spleen invasion** | 2.341[1.354-4.047] | **0.002** | 1.546[0.813-3.941] | 0.184 |
| **Marrow invasion** | 1.538[0.726-3.257] | 0.261 |  |  |
| **Central invasion** | 3.024[1.198-7.635] | **0.019** | 1.484[0.509-4.325] | 0.469 |
| **EB virus infection** | 0.563[0.320-0.990] | **0.046** | 0.551[0.284-1.069] | 0.078 |
| **PTGDS positive** | 1.116[1.031-1.207] | **0.006** | 1.261[1.122-1.417] | **<0.001** |

Abbreviations: IPI, international prognostic index; ESR, erythrocyte sedimentation rate; ALB, albumin; EB, Epstein-Barr; HR, hazard ratio.
