# Supplementary material for: Targeting PTGDS Promotes ferroptosis in peripheral T cell lymphoma through regulating HMOX1-mediated iron metabolism
Source: Br J Cancer. 2024 Dec 20;132(4):384–400. doi: 10.1038/s41416-024-02919-w (PMC11833084; doi:10.1038/s41416-024-02919-w)
Supplement: Supplementary file 7 — Supplementary materials and figures [file 41416_2024_2919_MOESM7_ESM.docx]

**Targeting PTGDS Promotes Ferroptosis in Peripheral T Cell Lymphoma through Regulating HMOX1-mediated Iron Metabolism** Shunfeng Hu^1,2^, Bingyu Liu^1^, Juanjuan Shang^1^, Qianqian Guo^2^, Tiange Lu^1^, Xiaoli Zhou^2^, Xiangxiang Zhou^2,3,4,5*^, Xin Wang^1,2,3,4,5*^

^1^ Department of Hematology, Shandong Provincial Hospital, Shandong University, Jinan, Shandong, 250021, China.

^2^ Department of Hematology, Shandong Provincial Hospital Affiliated to Shandong First Medical University, Jinan, Shandong, 250021, China.

^3^ Taishan Scholars Program of Shandong Province, Jinan, Shandong, 250021, China.

^4^ Branch of National Clinical Research Center for Hematologic Diseases, Jinan, Shandong, 250021, China.

^5^ National Clinical Research Center for Hematologic Diseases, the First Affiliated Hospital of Soochow University, Suzhou, 251006, China.

^*^**Corresponding authors**

Xin Wang, M.D., Ph.D.

Director & Professor of Department of Hematology,

Shandong Provincial Hospital, Shandong University

Add: No.324, Jingwu Road, Jinan, Shandong, 250021, China

Tel: 0086-531-68776358(B); 0086-13156012606(M)

Fax: 0086-531-87061197(B);

Email: [xinw007@126.com](mailto:xinw007@126.com)

Xiangxiang Zhou, M.D., Ph.D.

Department of Hematology, Shandong Provincial Hospital Affiliated to Shandong First Medical University

Add: No.324, Jingwu Road, Jinan, Shandong, 250021, China.

Tel: 0086-531-68776358(B); 0086-15866695595(M)

E-mail: [xiangxiangzhou@sdu.edu.cn](mailto:xiangxiangzhou@sdu.edu.cn)

**Conflict of Interest Disclosures**

The authors declare no competing financial interests.

Supplemental Materials and Methods

**Immunohistochemistry (IHC) and hematoxylin–eosin (HE) staining**

Fresh tissue were fixed with 4% paraformaldehyde and then processed by paraffin embedding. Sections (4 μm thick) were cut from formalin-fixed paraffin-embedded tissue blocks, which were further dewaxed and rehydrated. For antigen retrieval, sections were heated in 0.01 mol/L sodium citrate buffer (pH 6.0) under high pressure, followed by incubation with 3% solution of hydrogen peroxide to block endogenous peroxidases and normal goat serum to bock non-specific binding sites. Then, sections were incubated with specific primary antibodies overnight at 4 °C and second antibody for 1 hour at 37°C, followed by further incubation with strept avidin-horseradish peroxidase complex (SABC) and 3,3′- diaminobenzidine tetrachlorhydrate dehydrate (DAB). Finally, sections were counterstained with hematoxylin, mounted and photographed. Results of IHC were independently assessed by two observers at two different time points, who were blinded to patients’ information. IHC score was computed by the following formula: IHC score = proportion score (0, none; 1, 1%~25%; 2, 26%~50%; 3, 51%~75%; and 4, 76%~100%) × intensity score (0, negative; 1, weak; 2, moderate; and 3, strong). Scores of 0-7 were defined as negative expression, 8-12 as positive expression. The primary antibodies included PTGDS (ab182141, Abcam) and Ki67 (27309-1-AP, Proteintech Group).

**Flow cytometry analysis**

Following indicated treatment, PTCL cells were harvested and washed with PBS buffer. To evaluate cell cycle, PTCL cells were fixed with 70% ethanol overnight at -20 ℃ and stained using Propidium iodide (PI)/RNase Staining Buffer (550825, BD Biosciences, MA, USA) for 15-30 minutes. In cell apoptosis assay, PTCL cells were resuspended in 1 × binding buffer and incubated with Annexin V-PE/7-aminoactinomycin D (7AAD) (559763, BD Biosciences). To evaluate the level of lipid ROS production, PTCL cells were harvested and resuspended in 500 μL PBS buffer containing 2 μM C11-BODIPY (581/591) (D3861, Invitrogen). After incubation for 30 minutes at 37 °C, PTCL cells were then resuspended in 500 μL of fresh PBS buffer. Fluorescence was detected after gentle vibration and incubation in the dark at room temperature, using Navios Flow Cytometer (Beckman Coulter, CA, USA). Data were processed using FlowJo (FlowJo, LLC) software.

**Western blotting**

Total proteins were extracted from PTCL cells, normal T cells and mice tumor tissues using RIPA lysis buffer containing 1% protease inhibitor cocktail and 1 × phosphatase inhibitor cocktail (PhosSTOP, Roche, Basel, Switzerland). Then, the concentration of total protein was determined through BCA assay (Shenergy Biocolor), and 30 μg proteins were used for SDS-PAGE electrophoresis. Total proteins were transferred onto PVDF membrane (Millipore, MA, USA), which was incubated for 1 hour at room temperature in blocking solution. Following the incubation with primary antibodies at 4℃ overnight, membranes were washed in TBST, incubated with HRP-conjugated secondary antibody (Zhongshan Goldenbridge) for 1 hour at room temperature. Signals were detected using chemiluminescence detection reagent (Merck Millipore, MA, USA) on Amersham Imager 600 imaging system (General Electric, USA), which was analyzed via ImageJ software (NIH). The primary antibodies included HMOX1 (66743-1-Ig, Proteintech Group), NRF2 (66504-1-Ig, Proteintech Group), NCOA4 (39896, Proteintech Group), antibodies from Abcam, including PTGDS (ab182141), KEAP1 (ab227828), ACSL4 (ab155282), xCT (ab175186), GPX4 (ab125066), FTH1 (ab75972), FTL (ab109373), Ferritin (ab75973), P62 (ab109012), Beclin 1 (ab207612), LC3B (ab192890), and antibodies bought from Cell Signaling Technology (Beverly, USA), including c-myc (18583), Cyclin D1 (2922), CDK4 (12790), P21 (2947), P27 (3686), caspase 3 (9662), caspase 9 (9508), PARP (9532), Bax (5023), zeb1(3396), vimentin (5741) and HA (3724). β-tubulin (86298), β-actin (4967) and GAPDH (97166) were used as the internal reference.

**Quantitative real-time PCR**

RNAiso Plus reagent (Takara, Dalian, China) was used to extract total RNA from PTCL cells with indicated treatment, and PrimeScript RT reagent kit with gDNA eraser (Takara) was used for reverse transcription. Then, we used SYBR Green Master Mix (Takara) to evaluate the expression level of specific mRNA in LightCycler 480II real-time PCR system (Roche, Basel, Swizerland). The sequences for primers were as follows: PTGS2 forward, 5′-TCCCTTGGGTGTCAAAGGTAAA-3′; PTGS2 reverse, 5′-TGGCCCTCGCTTATGATCTG-3′; ATG2A forward, 5′-CCCAGCAAGGAGGTCTACGA-3′; ATG2A reverse, 5′-TGGGGTGAGATCAAAGCAGT-3′; HSPB1 forward, 5′-GCTTCACGCGGAAATACACG-3′; HSPB1 reverse, 5′-GTGATCTCGTTGGACTGCGT-3′. GAPDH was used as internal reference. Finally, the 2−ΔΔCt method was employed to compute the relative quantification of gene expression.

**Iron assay**

Ferrous iron level was determined using iron assay kit (ab83366, Abcam) according to the manufacturer’s instructions. PTCL cells with indicated treatment and tumor tissue from mice were washed twice using PBS buffer. After addition of iron assay buffer, ultrasonic cell-crushing device was used for cell disruption, and the supernatant was obtained by centrifugation. Then, samples were incubated at 37°C for 30 minutes in 96-well plates, and 100 µL iron probes were added into each well followed by the incubation at 37°C for 60 minutes protected from light. The absorbance values at 593 nm were measured, and the standard curves were drawn according to the absorbance values of standard samples. Ferrous iron level of samples was measured according to absorbance values and standard curves.

**Immunofluorescence assays and confocal microscopy**

PTCL cells with indicated treatment were spun onto cytospin glass slides and fixed using 4% paraformaldehyde for 15 minutes, followed by incubation with 0.1% Triton X 100 for 10 minutes. After blocking with 5% normal goat serum for 1 hour, primary antibodies were applied overnight at 4 °C and secondary antibodies for 1 hour at 37 °C. Then, slides were washed with PBS buffer, stained using DAPI and mounted. Confocal microscopy was performed using Leica TCS SP8 MP confocal microscope system (Germany). The primary antibodies included HMOX1 (66743-1-Ig, Proteintech Group) and PTGDS (ab182141).

**Co-immunoprecipitation (Co-IP)**

Total proteins were extracted from PTCL cells using Co-IP lysis buffer, which were incubated with 1-3 μg primary antibody at 4 °C overnight. Then, protein A/ G PLUS Agarose beads (Santa Cruz Biotechnology, USA) were added into lysis buffer, followed by the incubation at 4 °C for 2 hours. After washing for 3 times and the addition of loading buffer, the beads binding proteins were boiled at 100 °C for 10 minutes. Next, western blotting was performed to detect the expression of specific protein. The primary antibodies included PTGDS (ab182141, Abcam), HMOX1 (66743-1-Ig, Proteintech Group) and HA (2367, CST).

***In-vivo* xenograft tumor models**

4-week-old female severe combined immunodeficiency (SCID) beige mice were bought from Weitong Lihua Laboratory Animal Center (Beijing, China) and raised in specific pathogen-free (SPF) environment. All animal experimental were approved by Animal Care and Research Advisory Committee of Shandong Provincial Hospital. 1 × 10^7^ Karpas299 cells (untransfected, sh-Control, sh-PTGDS) were subcutaneously injected into the right hind legs of mice. Mice were divided into groups using simple randomization and researchers responsible for data collection remained blinded to randomization group. AT56 was dissolved in 0.5% methylcellulose and orally administered by gavage (80 mg/kg) while Sorafenib was dissolved in solvent containing PEG300 (topscience), Tween80 (topscience) and ddH_2_O, and intraperitoneally administered (80 mg/kg). The size of tumors was measured every day and tumor volume was calculated by the equation: V = (largest dimension × the perpendicular diameter ^2^) × 0.5. *In-vivo* small animal imaging system (Berthold Technologies, Germany) was used to evaluate the growth and metabolic activity of tumor. After 12-15 days, the mice were sacrificed and tumor tissues were collected, weighed, and processed for further experiments.

**Supplemental Figures**


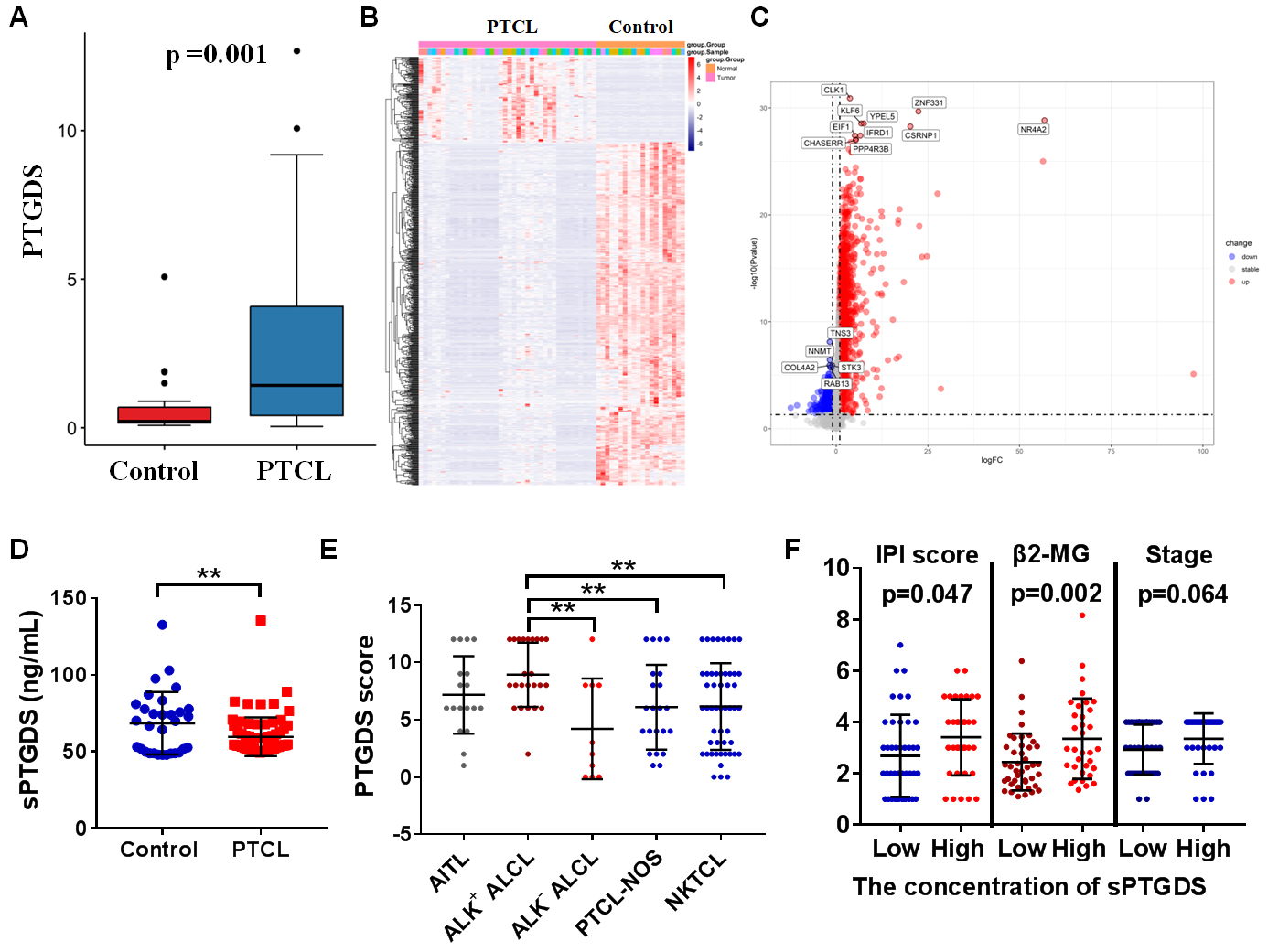


**Supplemental Figure 1. PTGDS expression was associated with clinical features in PTCL patients.** A. Analysis based on GSE6338 (PTCL patients, n= 40; normal T cells, n=20) showed the high expression of PTGDS in PTCL. B-C. The heatmap and volcano plot of differential expression genes were shown. D. The concentration of serum PTGDS was lower in PTCL patients (n = 76) than that in healthy control (n = 31). E. The expression of PTGDS was higher in ALK^+^ ALCL patients. F. The concentration of serum PTGDS was associated with clinical features in PTCL patients. Data are shown as the mean ± SD. **p < 0.01


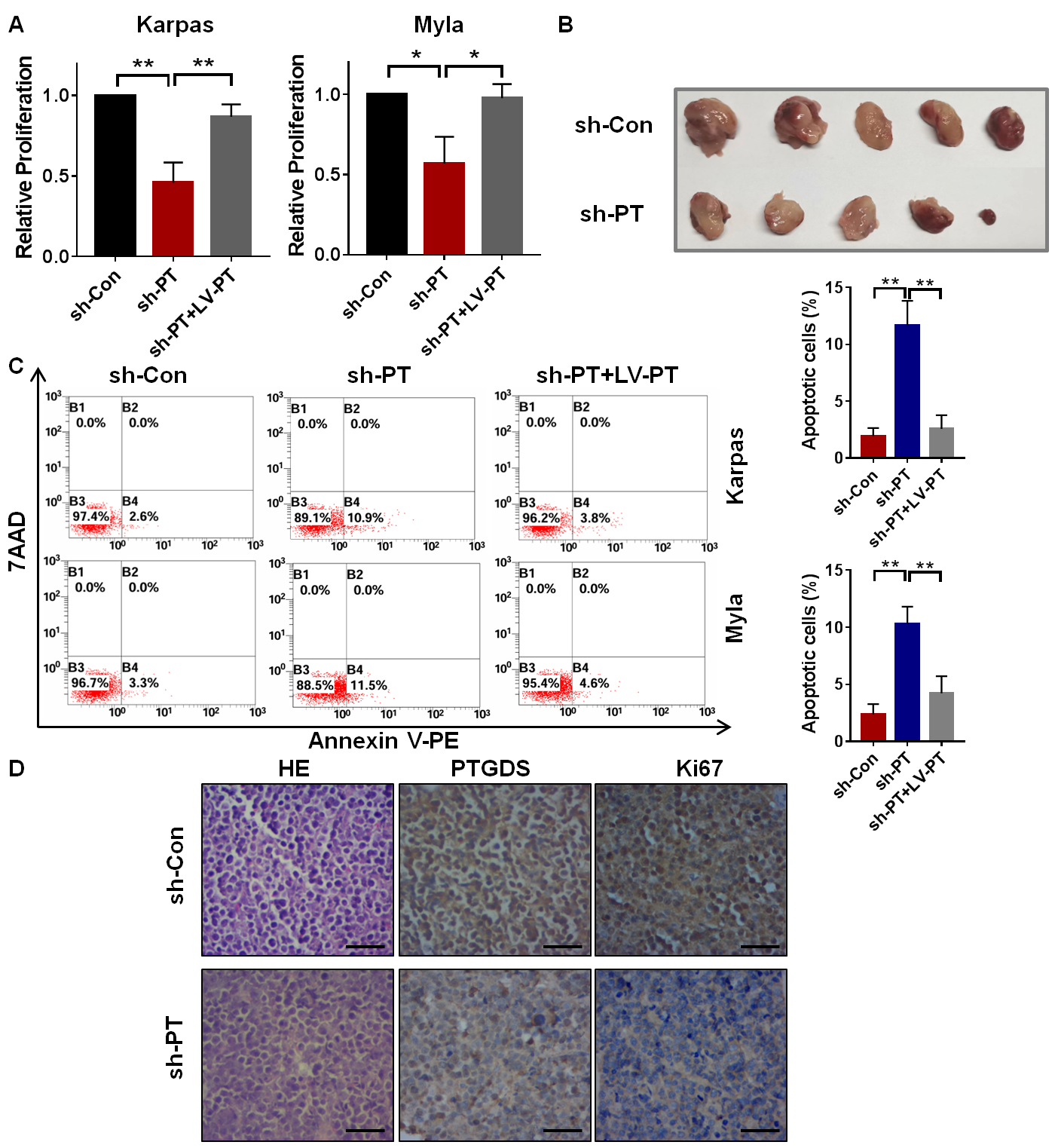


**Supplemental Figure 2. PTGDS knockdown inhibited the growth of PTCL cells*.*** A. PTGDS overexpression reversed the inhibitory effects of PTGDS knockdown on cell proliferation in PTCL. B. The pictures of tumor from PTCL mouse model. C. The promoting effects of PTGDS knockdown on cell apoptosis was partly reversed by PTGDS overexpression. D. Representative images of HE and IHC staining with PTGDS and Ki67 in xenograft tumor tissues. Bar = 50 μm.


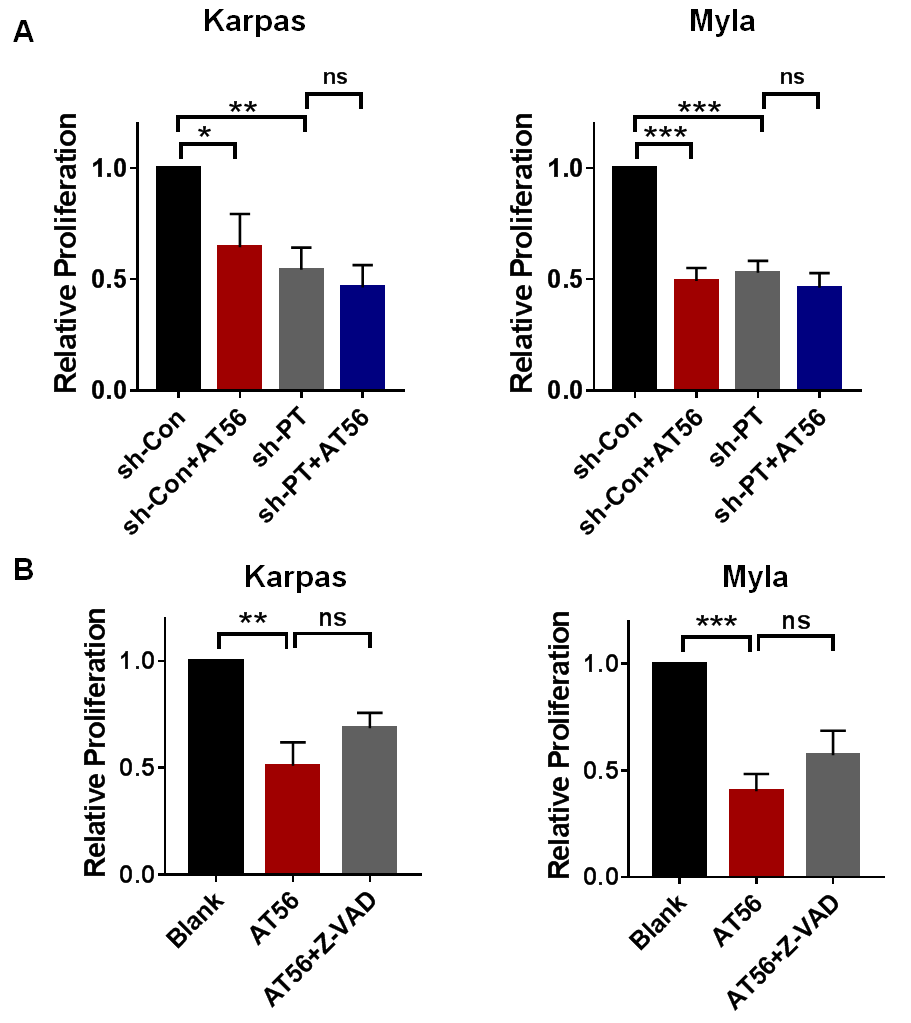


**Supplemental Figure 3. PTGDS inhibitor AT56 inhibited the proliferation of PTCL cells.** A. AT56 treatment inhibited cell proliferation in sh-Con cells, but not in sh-PTGDS cells. B. Apoptosis inhibitor, Z-VAD, partly rescued the anti-proliferation effects of AT56 treatment in PTCL cells, and the difference was not statistically significant. Data are shown as the mean ± SD. *p < 0.05; **p < 0.01; ***p < 0.001.


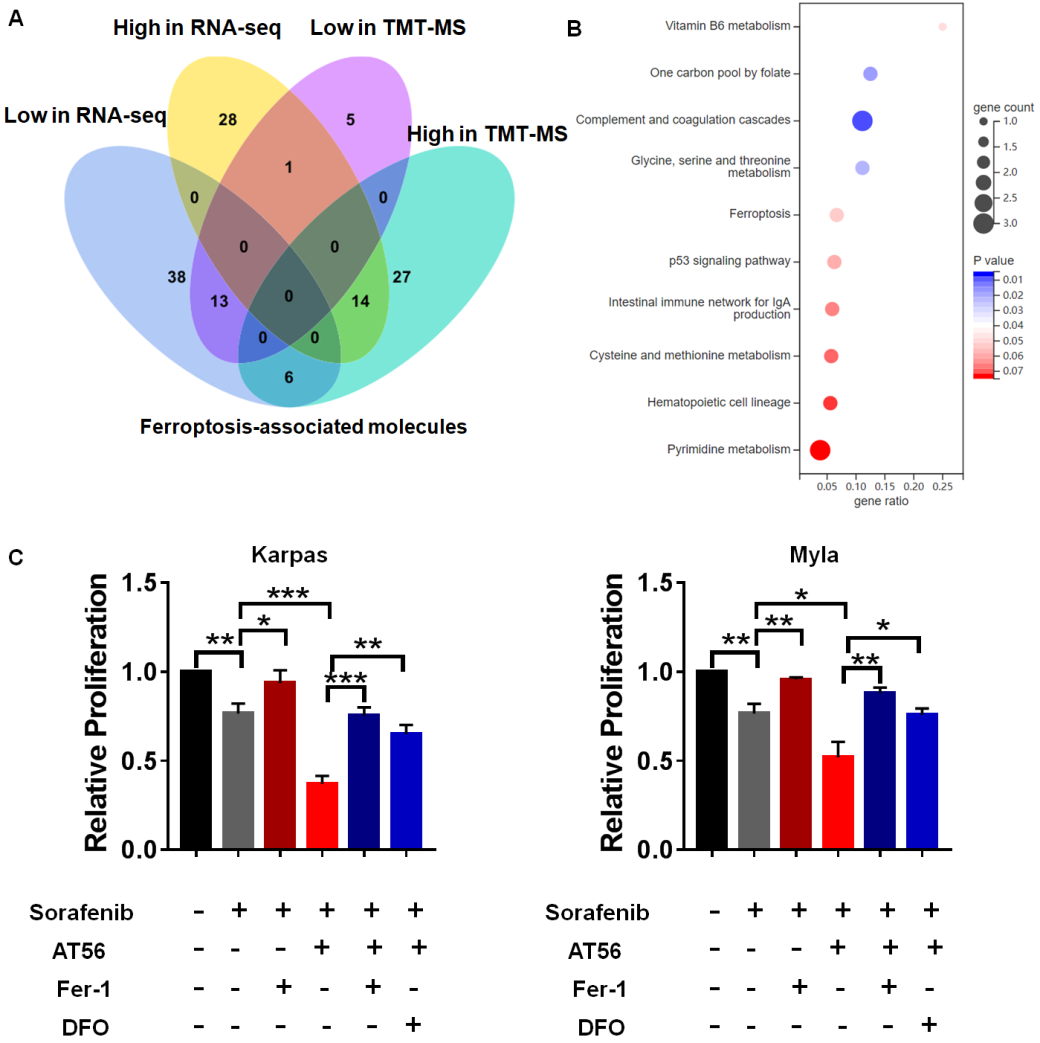

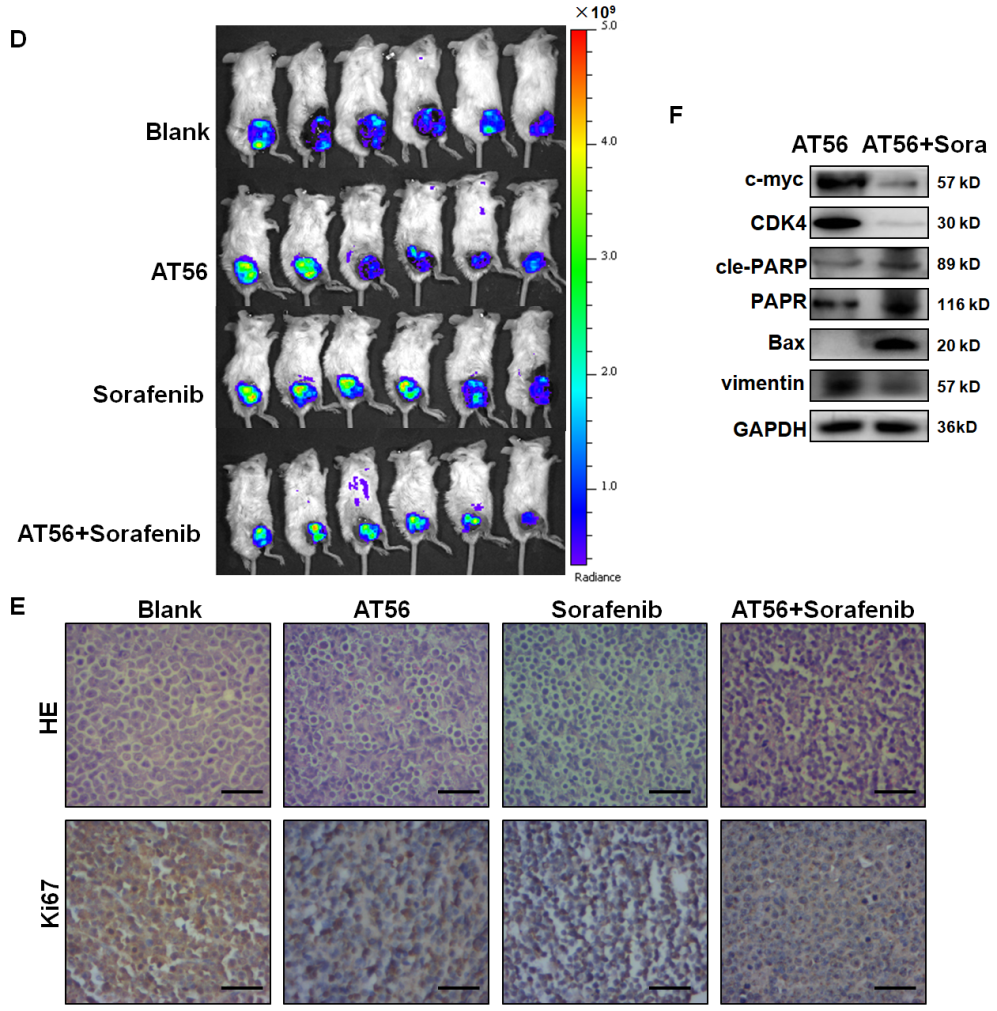


**Supplemental Figure 4. AT56 enhanced the inhibitory effects of Sorafenib on tumor growth in PTCL.** A. Venn diagram showed the overlap between up- and down-regulated ferroptosis-associated molecules in RNA-seq and TMT-mass spectrometry. There were 27 (low, n=13; high, n=14) molecules with consistent expression change trend in RNA-seq and TMT-mass spectrometry. B. KEGG pathway analysis based on the differentially expressed proteins (Fold change > 1.5, p < 0.05) in TMT mass spectrometry. C. The combined anti-tumor effects of Sorafenib and AT56 was reversed by Fer-1 and DFO. D-E. *In-vivo* imaging and IHC assay showed that the combination of AT56 and Sorafenib significantly inhibited tumor growth and Ki67 expression in PTCL. Bar = 50 μm. F. AT56 treatment enhanced the regulatory role of Sorafenib on the expression of important proteins in tumor tissues from mouse model.


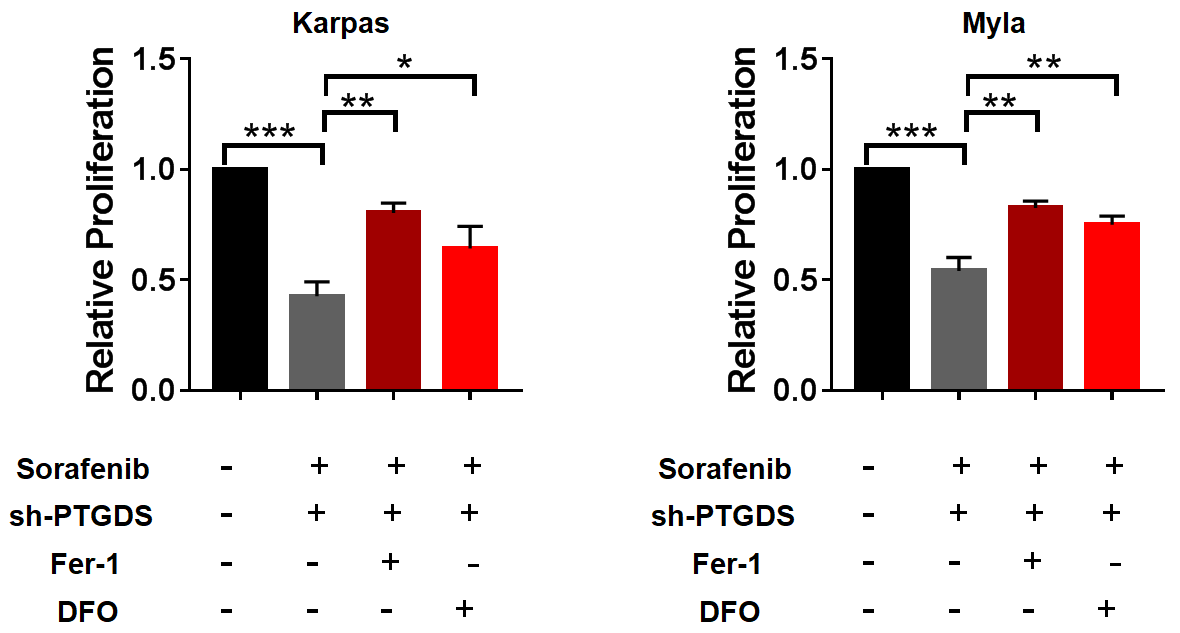


**Supplemental Figure 5. The combined anti-tumor effects of Sorafenib and PTGDS knockdown was dependent on ferroptosis process.** Fer-1 and DFO reversed the combined anti-tumor effects of Sorafenib and PTGDS knockdown in PTCL cells.


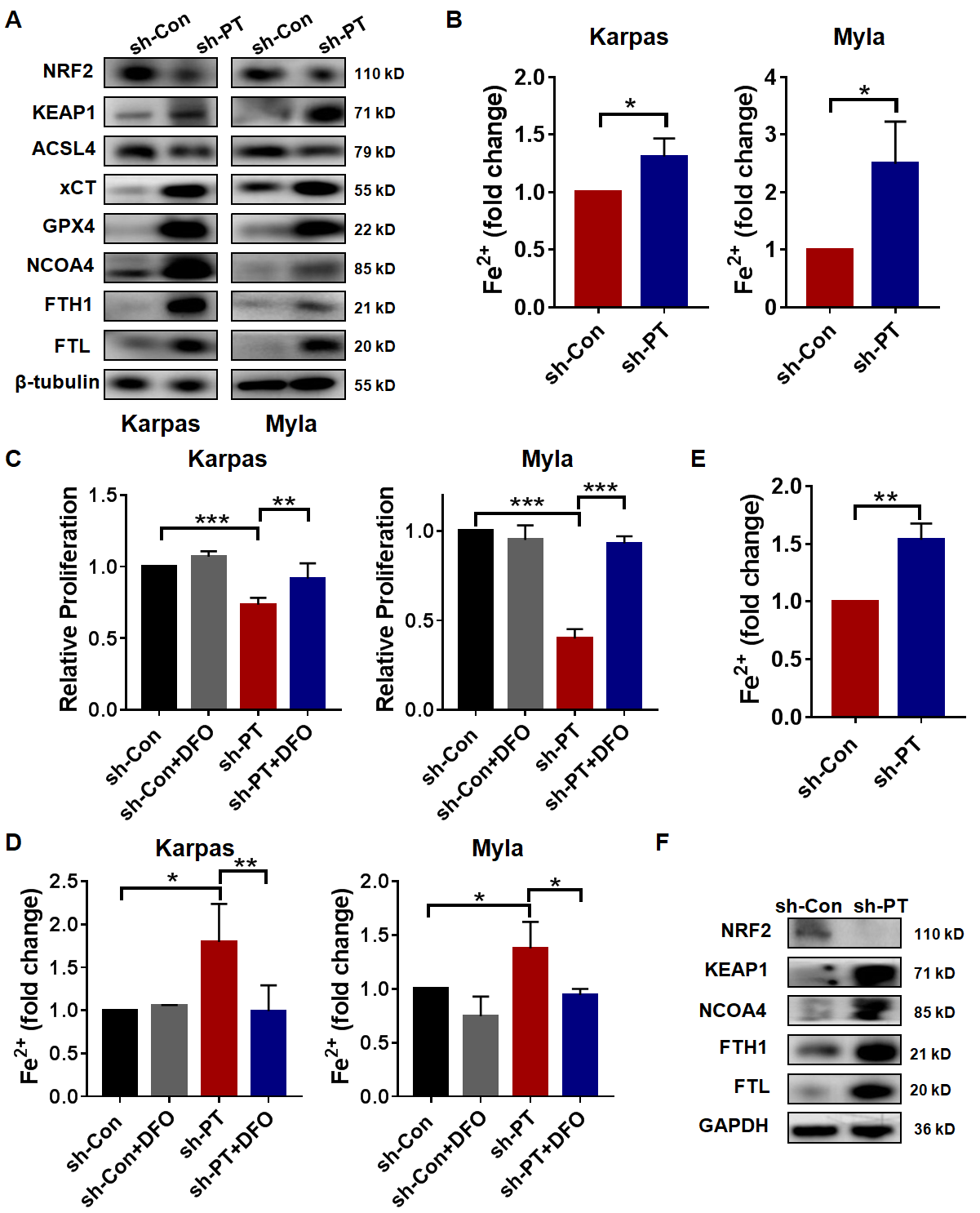


**Supplemental Figure 6. PTGDS knockdown promoted ferroptosis process through regulating iron metabolism in PTCL.** A. Western blotting results showed the expression level of ferroptosis-associated proteins in PTCL cells with PTGDS knockdown. B. PTGDS knockdown increased the level of Fe^2+^ in PTCL cells *in vitro.* C-D. DFO treatment reversed the proliferation inhibition and the accumulation of Fe^2+^ in PTCL cells with PTGDS knockdown. E. PTGDS knockdown increased the level of Fe^2+^ in PTCL tissue *in vivo*. F. Western blotting results showed the expression level of ferroptosis-associated proteins in tumor tissue from mouse model. Data are shown as the mean ± SD. *p < 0.05; **p < 0.01; ***p < 0.001.


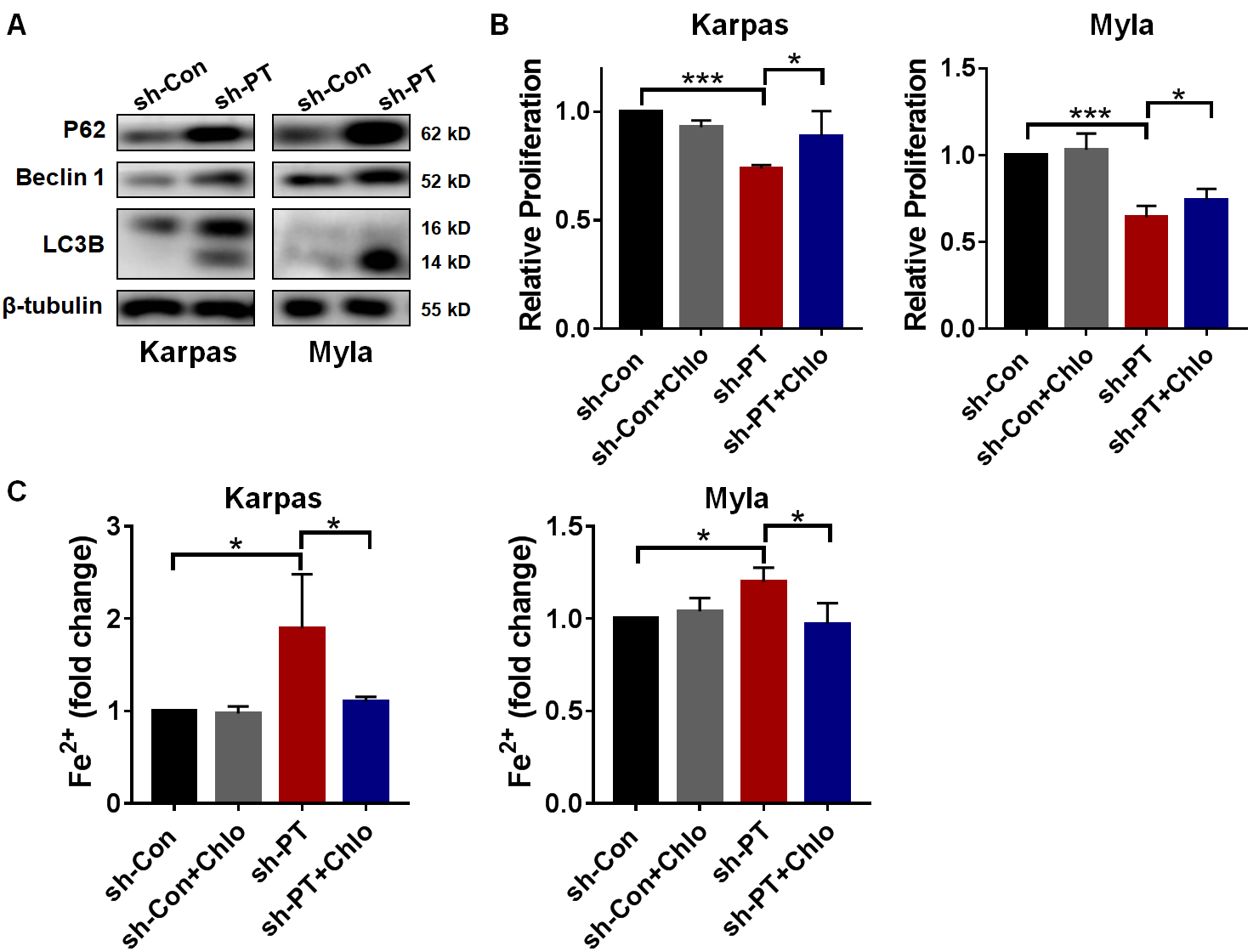


**Supplemental Figure 7. Targeting PTGDS promoted iron accumulation and ferroptosis through inducing ferritin autophagy in PTCL.** A. PTGDS knockdown increased the expression of P62, Beclin 1 and LC3B in PTCL cells. B. Chloroquine treatment partly reversed the inhibitory role of PTGDS knockdown on PTCL cell proliferation. C. The accumulation of Fe^2+^ caused by PTGDS knockdown was reversed by chloroquine treatment in PTCL cells. Data are shown as the mean ± SD. *p < 0.05; ***p < 0.001.


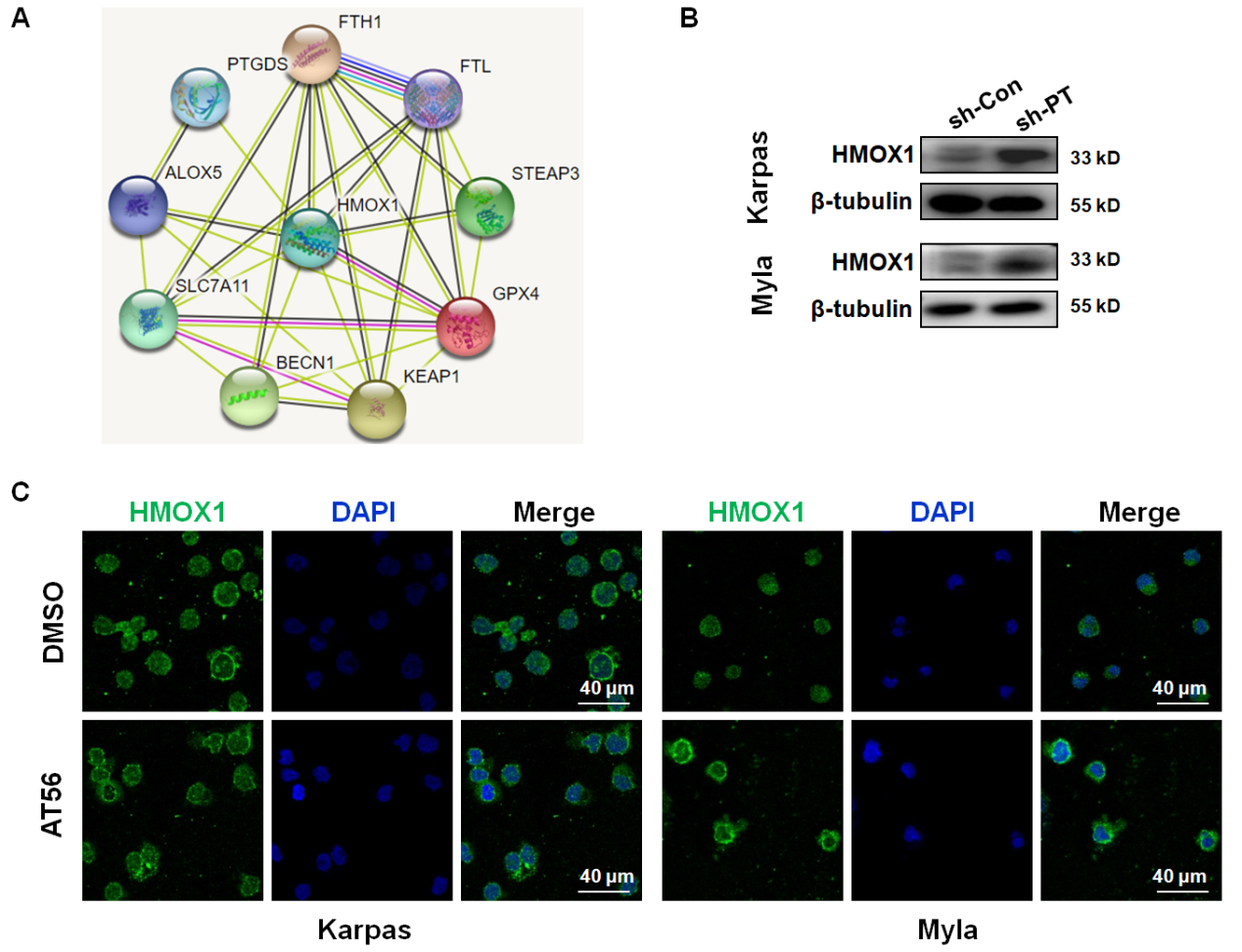


**Supplemental Figure 8. PTGDS interacted with HMOX1 and inhibited its expression in PTCL cells.** A. The potential interactions of PTGDS and differentially expressed ferroptosis-associated molecules. B. PTGDS knockdown increased the expression level of HMOX1 in PTCL cells. C. AT56 treatment had no effect on the intracellular localization of HMOX1 proteins. Bar = 40 μm
